# Colorectal cancer-derived osteopontin rewires macrophages into a pro-metastatic M2 state via the PI3K/AKT/CSF1-CSF1R axis

**DOI:** 10.1038/s41420-026-02945-y

**Published:** 2026-02-05

**Authors:** Xiaoxia Liang, Fei Qin, Ze Yuan, Minshan Wu, Jiawei Zhang, Xiaoxia Liu, Dianke Chen

**Affiliations:** 1 Department of Medical Oncology, Department of General Surgery, The Sixth Affiliated Hospital, Sun Yat-sen University, Guangzhou, Guangdong China; 2 Guangdong Provincial Key Laboratory of Colorectal and Pelvic Floor Diseases, The Sixth Affiliated Hospital, Sun Yat-sen University, Guangzhou, Guangdong China; 3 Biomedical Innovation Center, The Sixth Affiliated Hospital, Sun Yat-sen University, Guangzhou, Guangdong China; 4 State Key Laboratory of Oncology in South China, Sun Yat-sen University, Guangzhou, Guangdong China; 5 Department of Thoracic Surgery, Thoracic Cancer Center, The Sixth Affiliated Hospital, Sun Yat-sen University, Guangzhou, Guangdong China; 6 Guangdong Institute of Gastroenterology, Guangzhou, Guangdong China; 7 Key Laboratory of Human Microbiome and Chronic Diseases (Sun Yat-sen University), Ministry of Education, Guangzhou, China; 8 Department of Colorectal Surgery, Department of General Surgery, The Sixth Affiliated Hospital, Sun Yat-sen University, Guangzhou, Guangdong China; 9 Department of Gastrointestinal Surgery Section 2, Department of General Surgery, The Sixth Affiliated Hospital, Sun Yat-Sen University, Guangzhou, Guangdong China

**Keywords:** Colon cancer, Metastasis, Cancer microenvironment

## Abstract

Metastasis remains the primary cause of mortality in colorectal cancer (CRC), with a 5-year survival rate of ~14%, despite therapeutic advances. *SPP1*^+^ tumor-associated macrophages (TAMs) are implicated in promoting tumor progression, angiogenesis, and immune evasion. Osteopontin (OPN), encoded by the *SPP1* gene, is a critical regulator of TAMs M2 polarization and CRC metastasis when derived from TAMs. However, it remains unclear whether CRC-derived OPN interacts with M2-like TAMs to promote metastasis and what the underlying mechanisms are. Here, we found that OPN is highly expressed in metastatic CRC and is associated with poor prognosis. Contrary to prior reports, neither knockdown nor overexpression of OPN in CRC cells directly altered tumor cell invasion and migration. Rather, OPN expression levels were positively correlated with M2-like TAMs infiltration. The co-culture system revealed bidirectional chemotactic interactions between CRC cells-derived OPN and M2-like TAMs. Mechanistically, high OPN expression activates the PI3K/AKT signaling pathway in macrophages, promoting the secretion of CSF1, which induces M2-like polarization of macrophages to facilitate tumor metastasis. Finally, in a mouse metastasis model, blocking the CSF1/CSF1R axis with a CSF1R inhibitor reduced the M2-like TAMs recruitment and CRC tumor metastasis burden. Our study demonstrates that the OPN/PI3K/AKT/CSF1-CSF1R axis plays a crucial role in CRC metastasis. Blocking the CSF1/CSF1R axis reduces M2-like TAMs infiltration and tumor metastasis, offering a promising strategy for metastatic CRC.

## Introduction

Colorectal cancer (CRC) is the third most common malignancy globally and the second leading cause of cancer-related deaths, primarily due to metastasis [1]. Recent advancements in treatments such as surgery, radiotherapy, chemotherapy, molecular-targeted therapy, and immunotherapy have significantly improved the survival rates of CRC patients [2]. Despite these advancements, ~19.4–20.1% of these patients still develop distant metastases [3], leading to a 5-year survival rate of only 14% [4]. Therefore, it is essential to explore the mechanisms of CRC metastasis to identify potential therapeutic targets for these patients.

Cancer cells exist in a complex environment called the tumor microenvironment (TME), consisting of stromal cells, endothelial cells, and immune cells [5]. The TME is critical for tumor progression, therapeutic resistance, angiogenesis induction, and metastasis [6, 7]. Recent studies indicate that the absence of infiltrating immune cells in the TME is associated with poor prognosis in CRC patients [8, 9]. Tumor-associated macrophages (TAMs) at the tumor margin are thought to block cytotoxic T lymphocytes (CTLs) from entering the tumor core [10]. TAMs are a crucial component of the TME [11, 12] and can be polarized into classically activated M1 and alternatively activated M2 subtypes [13]. M1 inflammatory macrophages, activated by lipopolysaccharide (LPS) and interferon-γ (IFN-γ), produce high levels of reactive oxygen species (ROS) and can eliminate various pathogens and unwanted cells [14]. In contrast, M2-like macrophages promote tumor growth, angiogenesis, lymph node metastasis, and therapeutic resistance by secreting anti-inflammatory cytokines [15], matrix-degrading enzymes, and vascular endothelial growth factors [16–19].

Single-cell transcriptomic studies have identified a specific type of TAMs, termed *SPP1*^+^ TAMs, known for their immunosuppressive properties [20]. In gastric cancer, the immunosuppressive microenvironment dynamically associates with the presence of *SPP1*^+^ TAMs during anti-PD-1 immunotherapy [21]. Additionally, *SPP1*^+^ TAMs can interact with cancer-associated fibroblasts to prevent lymphocyte infiltration into the tumor core [22]. A single-cell RNA sequencing (scRNA-seq) analysis of head and neck squamous cell carcinoma (HNSCC) reveals that tumor-specific *SPP1*^+^ TAMs are associated with poor prognosis. They promote HNSCC cell proliferation and migration by secreting cytokines like tumor necrosis factor-α (TNF-α) and interleukin-1β (IL-1β) [23]. Notably, macrophage polarization defined by CXCL9/*SPP1* expression ratios has emerged as a pan-cancer prognostic biomarker [24]. Studies specific to CRC- reveal that *SPP1*^+^ TAMs impair T-cell infiltration, contributing to immunotherapy resistance, and are enriched in metastatic tissues, where their abundance inversely correlates with patient survival [22].

Osteopontin (OPN), a secreted glycophosphoprotein encoded by the *SPP1* gene [25], is expressed in both tumor cells and TAMs [26]. Acting as a key bridge molecule in the TME, OPN facilitates a bidirectional crosstalk between cancer and immune cells. This interaction establishes a positive feedback loop that promotes immunosuppression, tumor progression, and therapy resistance, highlighting its broad therapeutic potential [27]. Studies indicate that OPN orchestrates immune evasion by modulating the infiltration and function of CD8^+^ T cells, regulatory T cells, and M2 macrophages [28–30]. While the autocrine role of OPN in directly enhancing the migration, invasion, and adhesion of CRC cells is well-documented [31, 32], evidence from other cancers, including hepatocellular carcinoma (HCC), esophageal squamous cell carcinoma and nasopharyngeal carcinoma, highlights its paracrine role in polarizing M2 macrophages and promoting tumor progression [29, 33–35]. However, the involvement of OPN in mediating such crosstalk within the CRC microenvironment remains an open question. In this study, we demonstrate the reciprocal chemotaxis between CRC-derived OPN and M2-like TAMs. Mechanistic investigations reveal that OPN promotes M2 polarization of TAMs and facilitates CRC metastasis via the PI3K/AKT/CSF1/CSF1R pathway. Furthermore, blocking CSF1R significantly inhibits the recruitment of M2-like TAMs and suppresses CRC metastasis. Our findings offer novel insights into the interaction between CRC cells and the TME, highlighting potential therapeutic targets for CRC treatment.

## Results

### High expression of OPN in CRC patients correlates with poor survival

**Fig. 1:**
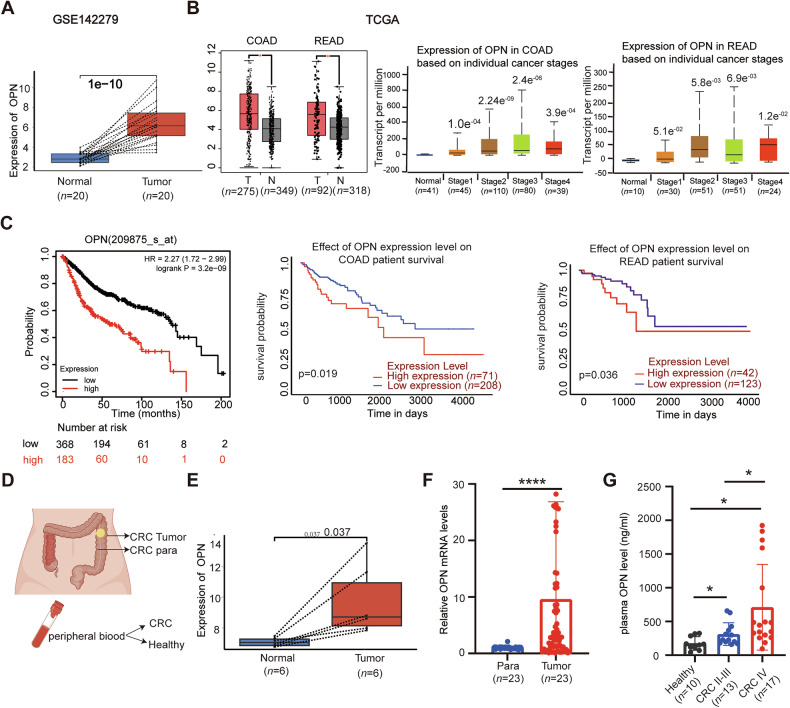
OPN is highly expressed in CRC patients and correlates with poor survival. **A** OPN expression in 20 paired CRC and adjacent tissues from the GSE142279 dataset (Deseq2). **B**, **C** TCGA data via UALCAN: **B** OPN expression in human CRC tumor tissues versus normal tissues (left), and correlation between OPN levels and CRC clinical stages (middle and right). **C** Kaplan–Meier curve illustrating the relationship between OPN expression and overall survival in CRC patients. **D**–**G** Validation in clinical specimens from our institution. **D** Schematic workflow: postoperative colorectal cancer tissues, adjacent tissues, and peripheral blood were collected from treatment-naive CRC patients (exclusion criteria: prior neoadjuvant therapy), and peripheral blood was collected from healthy donors. **E** Analysis of OPN in 6 paired CRC and adjacent tissues using Limma, from microarray data. **F** qPCR of OPN mRNA expression levels in 23 paired CRC and adjacent tissues. **G** ELISA analysis of OPN protein expression levels in peripheral blood from healthy individuals (*n* = 10) and CRC patients at stages II–III (*n* = 13) and stage IV (*n* = 17) CRC patients. All data are shown as mean ± SD; *p < 0.05; ****p < 0.0001; TCGA The Cancer Genome Atlas, COAD colon adenocarcinoma, READ Rectum adenocarcinoma.

To evaluate the clinical significance of OPN, we first analyzed its expression in two datasets: the GSE142279 dataset using Deseq2 (Fig. 1A) and in TCGA CRC samples through the Birmingham Cancer Data Analysis Portal (UALCAN) (Fig. 1B, C). The results showed that OPN expression levels were significantly higher in tumor tissues than in adjacent normal tissues and were significantly associated with higher TNM stages (Fig. 1A, B). Furthermore, Kaplan-Meier survival analysis conducted with UALCAN revealed that CRC patients with high OPN expression experienced significantly shorter overall survival (OS) compared to those with low OPN expression (Fig. 1C).

We further confirmed these findings using tissue samples and peripheral blood from CRC patients at our institution (Fig. 1D). Analysis using the limma package showed that OPN expression levels were higher in postoperative primary tumor tissues than in paired adjacent normal tissues. The results showed that OPN expression was higher in tumor tissues than in adjacent normal tissues (Fig. 1E). This finding was further validated by qPCR analysis, which yielded consistent results (Fig. 1F).

To investigate whether OPN expression levels are associated with metastasis, we performed an enzyme-linked immunosorbent assay (ELISA) to measure OPN levels in pre-treatment peripheral blood from patients with stage II-IV CRC and healthy individuals. The results showed that OPN expression levels were significantly higher in stage IV patients compared to healthy individuals and stage II–III patients (Fig. 1G). These findings suggest that OPN is significantly overexpressed in CRC and is associated with advanced disease stage and poor prognosis in patients.

### OPN is highly expressed in metastatic CRC

**Fig. 2:**
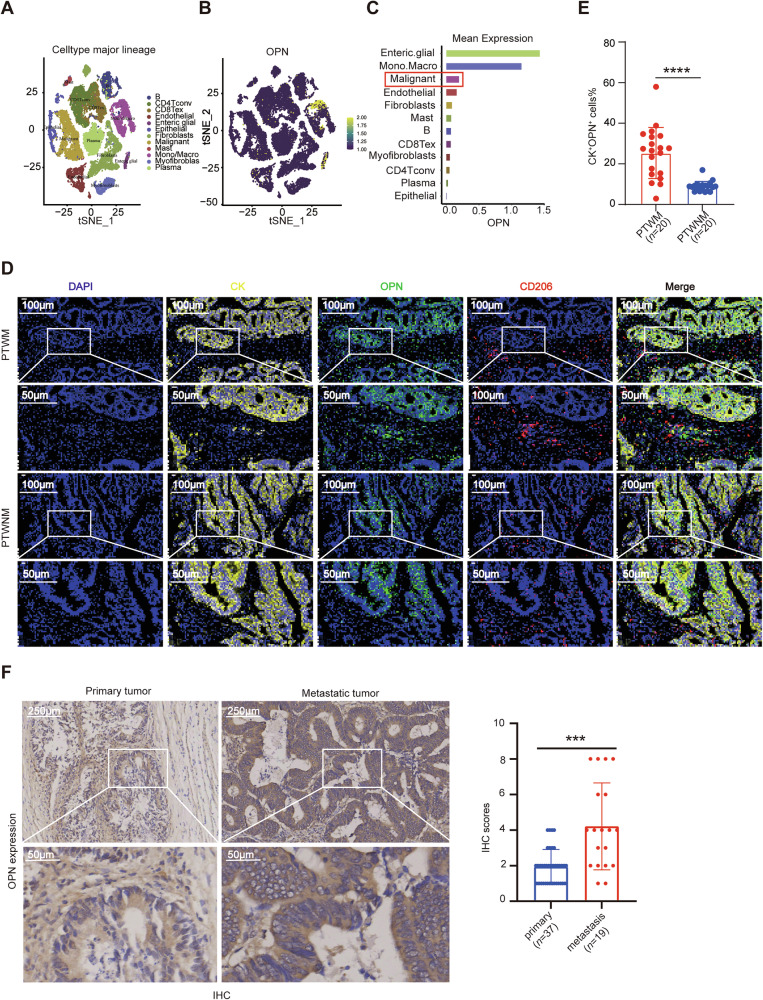
Cell type-specific expression patterns of OPN in CRC tissues. **A**–**C** Single-cell transcriptomic profiling of OPN expression in CRC tissues was performed using a publicly accessible database (https://www.aclbi.com/static/index.html#/single_cell_search). **A** Distribution of distinct cell types in CRC tumor tissues, revealed 12 clusters, each depicted by a unique color. **B** Mapping OPN expression at single-cell resolution across the 12 annotated clusters. **C** Bar graph depicting the mean expression levels of OPN in different cell types. **D**, **E** Multiplex immunohistochemistry (mIHC) analysis of OPN expression in CRC patients from our cohort. **D** Representative mIHC images co-staining OPN (green), tumor cells (cytokeratin [CK] marker, yellow), and TAMs (CD206 marker, red) in primary tumors with metastasis (PTWM, top) (*n* = 20) and primary tumors without metastasis (PTWNM, bottom) (*n* = 20), Scale bars: 200 μm (upper panels), 50 μm (lower panels). **E** Quantification of CK and OPN dual-positive tumor cells in PTWM versus PTWNM. **F** Representative images of IHC staining of OPN in primary (*n* = 37) and metastatic (*n* = 19) CRC tissues (left), along with statistical analysis of IHC scores of OPN presented as a bar graph (right). Scale bars: 250 μm (top images), 50 μm (bottom images). IHC immunohistochemistry, mIHC multiplex immunohistochemistry, TAMs tumor-associated macrophages. All data are shown as mean ± SD; ***p < 0.001; ****p < 0.0001.

To determine the spatial distribution of OPN within CRC tumors, we first analyzed a scRNA sequencing dataset from CRC patients (https://www.aclbi.com/static/index.html#/single_cell_search, CRC_EMTAB8107). The results revealed that OPN is highly expressed not only in TAMs but also in malignant epithelial cells (Fig. 2A–C). To further validate these findings, we performed multiplex immunohistochemistry (mIHC) on clinical samples of primary CRC tumors, both with (*n* = 20) and without (*n* = 20) distant metastases. Strikingly, primary tumors with metastasis (PTWM) showed marked overexpression of OPN in both M2-like TAMs (CD206^+^) and CRC tumor cells (pan-CK). Moreover, OPN expression levels in tumor cells showed a significant positive correlation with metastatic progression (Fig. 2D, E). Additionally, immunohistochemical (IHC) analysis of primary and metastatic tumor tissues from CRC patients revealed higher OPN expression in metastatic lesions (Fig. 2F). These findings indicate that OPN is highly expressed in CRC cells from primary tumors with metastases.

### The metastatic potential of CRC Cells is not determined by OPN expression Levels

**Fig. 3:**
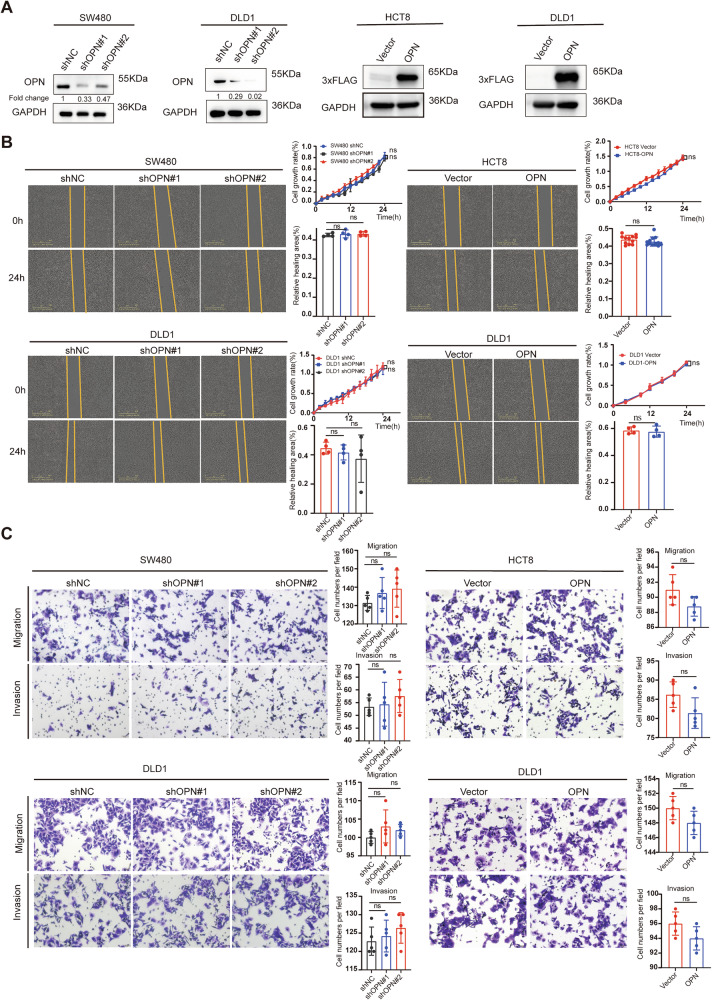
Tumor cell-secreted OPN is not a driver of CRC cells invasion and metastasis. **A** Western blot analysis of OPN shRNA (shOPN), control shRNA (shNC), OPN overexpression (OE), and FLAG-tagged OPN levels in human CRC cell lines, with GAPDH as the loading control. **B** Wound closure assays using the IncuCyte system to measure cell migration over 24 hours (left), with statistical analysis of wound closure (bottom right) and simultaneous cell proliferation analysis (top right). **C** Transwell assays to assess the effects of OPN knockdown (shOPN) and overexpression (OE) on CRC cells' migration (upper panels) and invasion (lower panels) capabilities. All data are shown as mean ± SD; ns, not significant.

The aforementioned results prompted us to further investigate how OPN expression levels in tumor cells affect CRC metastasis. qPCR analysis revealed no significant differences in the mRNA expression levels of the OPN splice variants (b, c, 4, and 5) [36] among HCT8, SW480, and DLD1 cells (Fig. S1). First, we employed a lentiviral expression system to knock down OPN in CRC cell lines DLD1 and SW480, and to overexpress OPN in DLD1 and HCT8 cells (Fig. 3A). Subsequently, we conducted proliferation and wound-healing assays using the IncuCyte system. The results showed that neither OPN knockdown nor overexpression significantly altered CRC cell proliferation or migration (Fig. 3B). Additionally, transwell invasion and migration assays revealed that OPN expression levels did not influence the invasion or migration of CRC cells, although there was a slight trend toward the opposite effect (Fig. 3C). These findings suggest that the invasion and metastasis of CRC cells are not directly dependent on tumor-derived OPN expression levels.

### OPN expression positively correlates with M2-TAMs infiltration

**Fig. 4:**
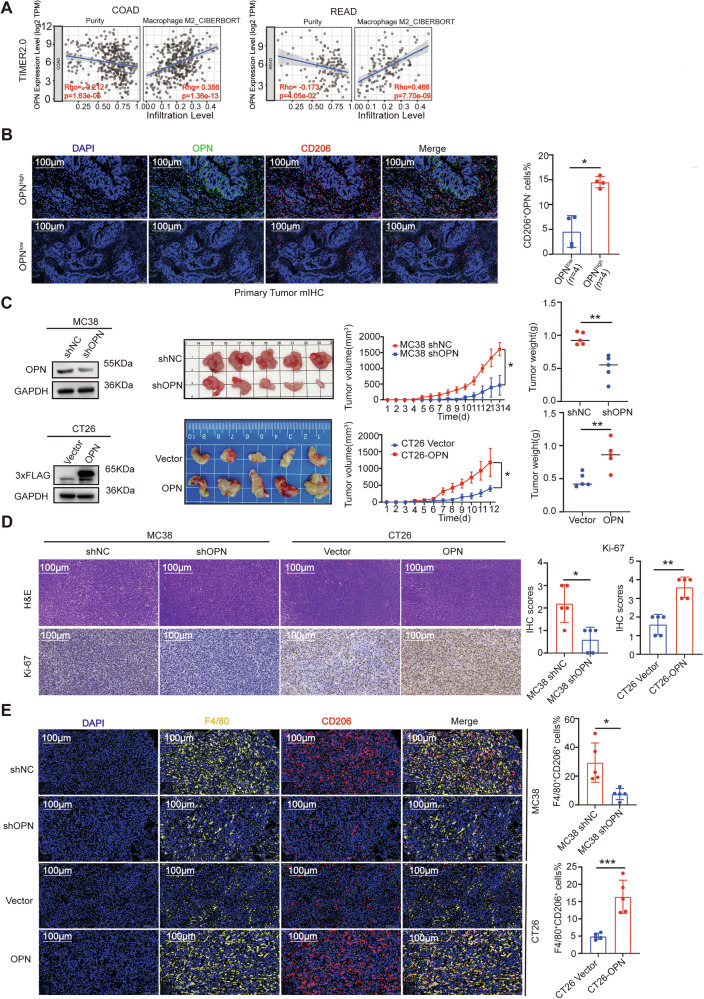
OPN expression positively correlates with M2-TAMs infiltration. **A** TIMER2.0 database analysis reveals a positive correlation between OPN and M2-TAMs infiltration in CRC tissues. **B** mIHC analysis of M2-TAMs (CD206, red) in human CRC primary tumor tissues with high (*n* = 4) and low (*n* = 4) OPN expression (OPN, green) (left). Statistical analysis of CD206 proportion (right). **C**–**E** Subcutaneous tumor models in BALB/c-nu mice injected with murine CRC cells with OPN knockdown (*n* = 5) or OPN overexpression (*n* = 5). **C** Western blot analysis of shOPN, shNC, OPN overexpression, and control FLAG-tag OPN levels in murine CRC cells (left). Tumor images, growth curves (two-way ANOVA test), and average tumor weight at sacrifice (right). **D** H&E (top) and Ki-67 (bottom) staining of tumor sections (left). Scale bar: 100 μm. Statistical analysis of Ki-67 IHC scores (right). **E** mIHC assessment of M2-TAMs in each group: Representative images co-staining for F4/80 and CD206 (left). Scale bar: 100 μm. Statistical analysis of F4/80^+^CD206^+^ proportion (right). All data are shown as mean ± SD; *p < 0.05; **p < 0.01; ***p < 0.001.

While our results indicated that the intrinsic expression level of OPN in CRC cells does not significantly affect CRC cell migration, this unexpected finding prompted further investigation into its interactions within the tumor stroma. Studies have shown that during tumor progression, M2-polarized phenotype TAMs are recruited to the tumor, where they regulate tumor cell activation, immune suppression, angiogenesis, and tumor metastasis [37]. Additionally, research has shown that *SPP1*^+^ TAMs are associated with tumor growth and metastasis [20]. Therefore, we hypothesized that M2-like TAMs in the TME may interact with tumor-derived OPN to influence tumor progression and metastasis. To test this hypothesis, we used the TIMER2.0 online tool to analyze the correlation between OPN expression levels and M2-like TAMs infiltration in CRC. The results revealed a positive correlation between OPN expression and M2-TAMs infiltration. Notably, tumor purity, defined as the proportion of malignant cells in the specimen, exhibited a significant inverse correlation with OPN expression, implying that elevated OPN levels are predominantly derived from non-neoplastic compartments and are positively associated with the abundance of M2-TAMs (Fig. 4A). Based on this observation, we next performed mIHC to examine the relationship between M2-like TAMs infiltration and OPN expression on primary CRC tumor tissues, which were classified into high (OPN^high^) and low (OPN^low^) OPN expression groups. The results demonstrated that OPN^high^ tissues exhibited higher levels of M2-like TAMs infiltration (Fig. 4B). To further explore whether OPN expression in CRC cells affects M2-like TAMs infiltration in the TME, we knocked down OPN in MC-38 cells and overexpressed OPN in CT26 cells. These cells, along with their respective control cells, were then implanted into BALB/c nude mice to establish subcutaneous xenograft models. As shown in Fig. 4C, D, OPN knockdown inhibited tumor growth, while OPN overexpression stimulated tumor growth. Further mIHC analysis of M2-TAMs markers (F4/80 and CD206) in OPN knockdown and OPN overexpression tumor tissues consistently revealed higher expression of M2-TAMs markers in the OPN overexpression group compared to the OPN knockdown group (Fig. 4E). These findings suggest that OPN derived from CRC tumors is positively correlated with increased M2-like TAMs infiltration.

### OPN induces M2-like polarization of macrophages and promotes mutual migration

**Fig. 5:**
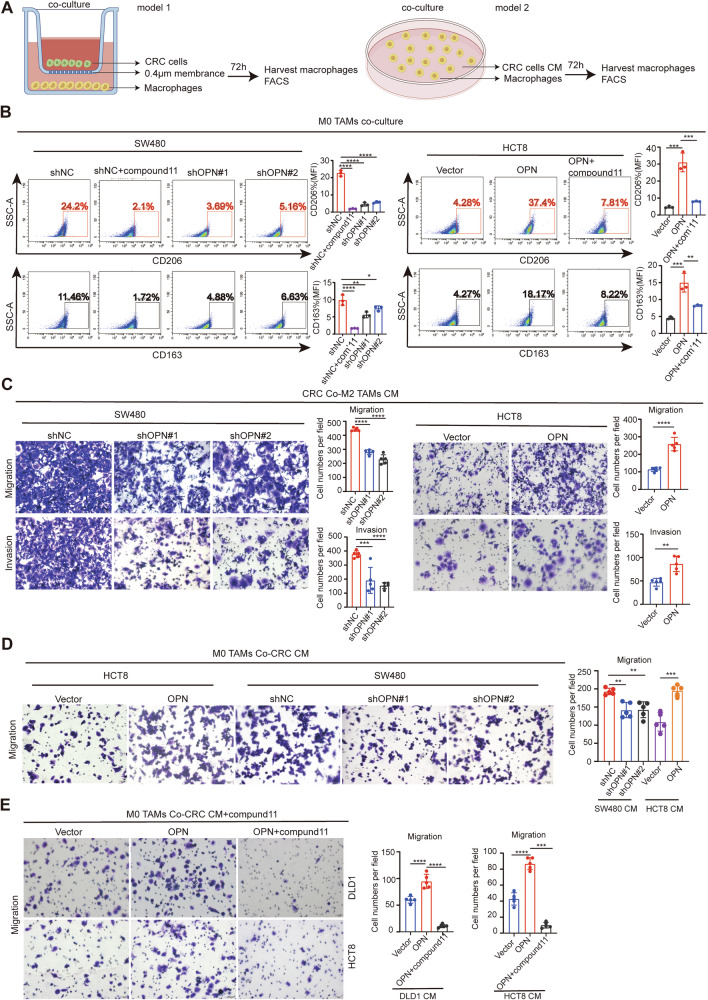
OPN induces M2-like polarization of macrophages in CRC and promotes reciprocal migration. **A** Experimental schematics of macrophage polarization models: indirect co-culture using Transwell chambers (0.4-μm pores) with CRC cells ± OPN inhibitor Compound 11(20 μM) (model 1, left); direct polarization via conditioned medium (CM) exposure(model 2, right). PMA-differentiated THP-1 macrophages were analyzed by flow cytometry after 72 h. **B** Flow cytometry analysis of CD206 (top) and CD163 (bottom) expression on macrophages. **C** Transwell migration and invasion assays of CRC cells (SW480, HCT8) co-cultured with M2-TAMs CM (left), with migrated CRC cells counts (right). **D** Transwell migration assays of PMA-stimulated THP-1 macrophages co-cultured with CRC cells CM (SW480, HCT8) (left), with migrated macrophage counts (right). **E** Transwell migration assays of PMA-stimulated THP-1 macrophages co-cultured with conditioned medium(CM) from OPN^high^ CRC cells treated with OPN inhibitor (20 μM) (DLD1, HCT8) (left), with quantitative migration results (right). All data are shown as mean ± SD; **p < 0.01; ***p < 0.001; ****p < 0.0001.

To further investigate the effect of tumor-derived OPN in CRC on M2-like polarization of macrophages and their mutual chemotactic migration, we established two in vitro co-culture systems. In the first system, conditioned medium (CM) from CRC cells with different OPN expression levels was co-cultured with phorbol 12-myristate 13-acetate (PMA)-treated THP-1 cells (monocyte-derived macrophages induced from the THP-1 cell line) for 72 hours. In the second system, a 0.4 μm Transwell setup was used, where CRC cells with different OPN expression levels were placed in the upper chamber and PMA-treated THP-1 cells in the lower chamber for 72 hours. The impact of tumor-derived OPN expression on M2-like polarization of TAMs was then evaluated (Fig. 5A). Flow cytometry analysis revealed that macrophages co-cultured with OPN^high^ CRC cells (HCT8-OPN and SW480-shNC) exhibited higher expression levels of M2-like markers CD206 and CD163. The concentration of OPN in the CM ranged from 224 to 350 ng/mL (Fig. S3B). In contrast, macrophages co-cultured with OPN^low^ CRC cells (SW480-shOPN and HCT8-Vector) showed lower levels of these markers. Compound 11, a derivative of dihydroartemisinin (molecular formula C_25_H_33_N_3_O_5_), was obtained as a white-to-pale-yellow crystalline solid. Structurally, it incorporates a dihydroartemisinin (DHA)-ether core connected via a rigid 1,2,3-triazole linker that targets the hydrophobic groove essential for OPN transcriptional activation [38]. When OPN^high^ CRC cells (HCT8-OPN and SW480-shNC) were treated with compound 11 (20 μM, IC_50_ = 28.8 μM, Fig. S2) as an OPN inhibitor, co-cultured macrophages showed reduced expression of M2-like markers CD206 and CD163 (Fig. 5B). In contrast, the M1-like marker CD86 remained relatively unaltered (Fig. S3A), demonstrating its specific inhibitory effect on OPN-mediated M2 polarization. Building on these findings, we next explored whether the expression level of tumor-derived OPN in CRC and M2-like TAMs mutually influences chemotactic migration by performing Transwell migration assays. THP-1 cells were further induced into M2-like TAMs using PMA/IL-4/IL-13, and the supernatant (CM) from these M2-like TAMs was collected and co-cultured with CRC cells exhibiting varying levels of OPN expression. The results showed that the CM from M2-like TAMs CM promoted the invasion and migration of OPN^high^ CRC cells, including HCT8-OPN and SW480-shNC (Fig. 5C), as well as DLD1-OPN and DLD1-shNC (Fig. S3C). To investigate whether tumor-derived OPN in CRC also affects the chemotactic migration of macrophages, we conducted migration assays using PMA-treated THP-1 cells co-cultured with CRC cells with different OPN expression levels or with CM from OPN-overexpressing CRC cells treated with the OPN inhibitor. The results demonstrated that CM from OPN-overexpressing CRC cells (HCT8-OPN and SW480-shNC) induced chemotactic migration of macrophages (Fig. 5D). However, adding an OPN inhibitor to OPN^high^ CRC cells (HCT8-OPN and DLD1-OPN) attenuated the chemotactic migration ability of TAMs (Fig. 5E). Together, these findings suggest that tumor-derived OPN drives M2-like polarization of macrophages through secretory mechanisms and promotes mutual chemotactic migration between tumor cells and macrophages.

### OPN induces macrophage secretion of CSF1 via the PI3K/AKT axis

**Fig. 6:**
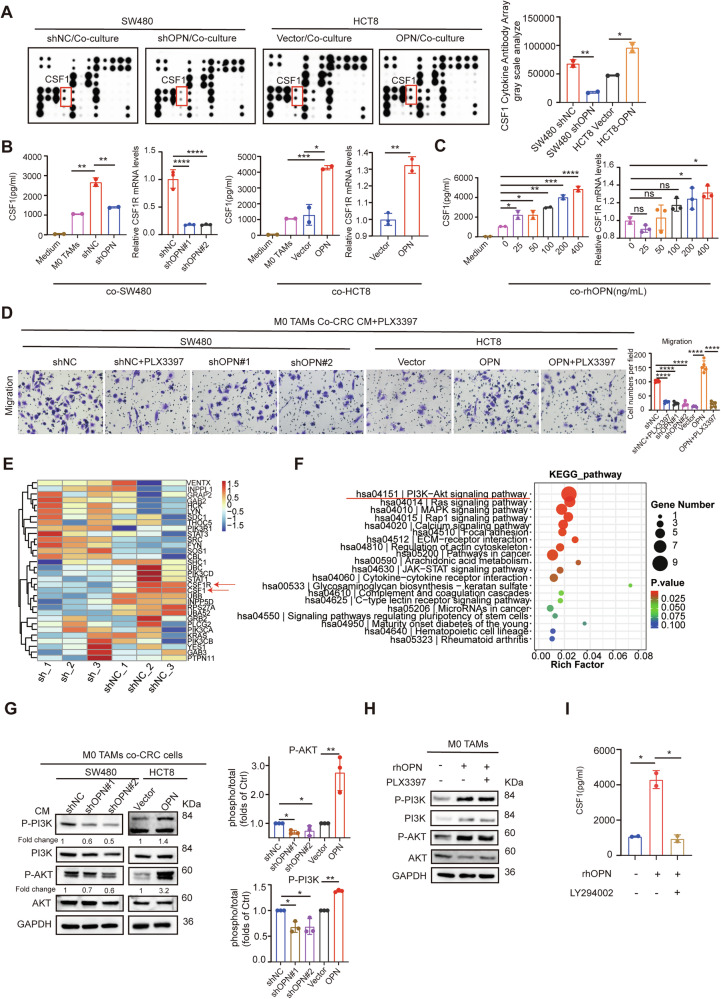
OPN activates the PI3K/AKT signaling axis to promote macrophage secretion of CSF1. **A** Cytokine array profiling of macrophage secretomes under OPN-modulated co-culture conditions: representative array membranes comparing OPN knockdown (shOPN) vs. overexpression (OE) in CRC-TAM co-cultures. (left) and quantification of CSF1 as the most differentially secreted factor. (right). **B** Macrophages treated with conditioned medium (CM) from CRC cells for 72 hours show CSF1 expression by ELISA (left) and CSF1R expression by qPCR (right). **C** Macrophages treated with rhOPN for 72 hours show CSF1 expression by ELISA (left) and CSF1R expression by qPCR (right). **D** Transwell migration assays of M0 macrophages or PLX3397-treated macrophages co-cultured with CRC cell CM (left), with summarized quantitative results (right). **E**, **F** RNA-seq of M0 macrophages co-cultured with SW480-shNC and SW480-shOPN#2 CM for 48 hours. **E** Heatmap of macrophage polarization-related genes (z-score analysis, upregulated in red). **F** KEGG analysis of enriched pathways. **G** Western blot of PI3K/AKT phosphorylation in macrophages cultured with CRC cells CM for 72 hours (left), with quantitative results (right). **H**–**I** Pharmacological perturbation of the signaling pathway hierarchy. **H** Western blot of PI3K(p85/p55)/AKT(Ser473) in macrophages pretreated with LY294002 (PI3K inhibitor, 10 μM) or PLX3397 (CSF1R inhibitor, 10 μM) followed by rhOPN (400 ng/ml) treatment for 48 hours. **I** CSF1 secretion was quantified by ELISA in macrophage supernatants under corresponding treatments. All data are shown as mean ± SD; *p < 0.05; **p < 0.01; ***p < 0.001; ****p < 0.0001; ns not significant.

Based on the aforementioned results demonstrating that CRC-derived OPN induces chemotactic migration of macrophages, we used the RayBio human cytokine antibody array to compare the cytokine profiles of macrophage-conditioned media to identify OPN-regulated secretomes. Comparative analysis revealed CSF1 as the most differentially secreted factor in OPN^high^ (SW480-shNC/HCT8-OPN) versus OPN^low^ (SW480-shOPN/HCT8-Vector) co-cultures (Fig. 6A). Previous studies have reported that CSF1 regulates macrophage differentiation, maintenance, and proliferation [39], and its receptor, CSF1R, is essential for maintaining TAMs [40]. Additionally, CSF1 expression has been shown to play a key role in metastatic tumors [41]. ELISA quantification validated these findings, showing that CSF1 levels were elevated in OPN^high^ CRC/macrophage co-cultures compared to OPN^low^ cells (Fig. 6B). Concomitant qPCR (Fig. 6B) and Western blot (Fig. S5A, B) analysis demonstrated CSF1R upregulation in macrophages following exposure to OPN^high^ tumor cells.

To establish whether OPN directly stimulates CSF1 secretion from macrophages, we treated these cells with recombinant human OPN protein (rhOPN). ELISA quantification of the co-culture supernatants revealed that rhOPN dose-dependently induced CSF1 secretion. This effect was further supported by consistent upregulation of CSF1R expression, as confirmed by qPCR (Fig. 6C) and Western blot (Fig. S5C), in macrophages treated with rhOPN. Pharmacological inhibition of CSF1R with PLX3397 (10 μM; Fig. S4) abolished OPN-driven macrophage migration, confirming dependency on the CSF1R pathway (Fig. 6D). Furthermore, combination treatment with PLX3397 and the OPN inhibitor compound 11 resulted in a more potent inhibition of macrophage migration compared to either agent alone (Fig. S5D, E).

To further elucidate the mechanism of OPN-mediated macrophage chemotactic migration, we performed RNA sequencing of M0 macrophages co-cultured with CM from SW480-shOPN- and shNC-transfected cells. The results revealed simultaneous upregulation of CSF1 and CSF1R, along with significant enrichment of the PI3K/AKT pathway (Fig. 6E, F). Moreover, western blot confirmed that the phosphorylation levels of PI3K and AKT were significantly elevated in macrophages co-cultured with OPN^high^ CRC cells (Fig. 6G). Similar expression patterns were observed in CT26-OPN (OPN^high^) subcutaneous tumors (Fig. S5F, G). Next, to delineate the causal relationship between PI3K/AKT activation and CSF1 secretion, we performed targeted pathway inhibition in rhOPN-stimulated macrophages. The results demonstrated that pre-treatment with the PI3K inhibitor LY294002 (10 μM) reduced OPN-induced CSF1 secretion, whereas CSF1R blockade with PLX3397 (10 μM) showed no significant effect on PI3K or AKT phosphorylation (Fig. 6H, I). Furthermore, pharmacological inhibition of PI3K markedly suppressed both the invasion and migration of CRC cells (Fig. S6A, B). Separately, it also inhibited the M2 polarization of macrophages (Fig. S6C, D). In summary, these findings demonstrate that OPN promotes the secretion of CSF1 by macrophages through the PI3K/AKT signaling axis, thereby enhancing chemotactic migration in CRC.

### Targeting CSF1R attenuates M2-TAMs recruitment and metastatic dissemination in vivo

**Fig. 7:**
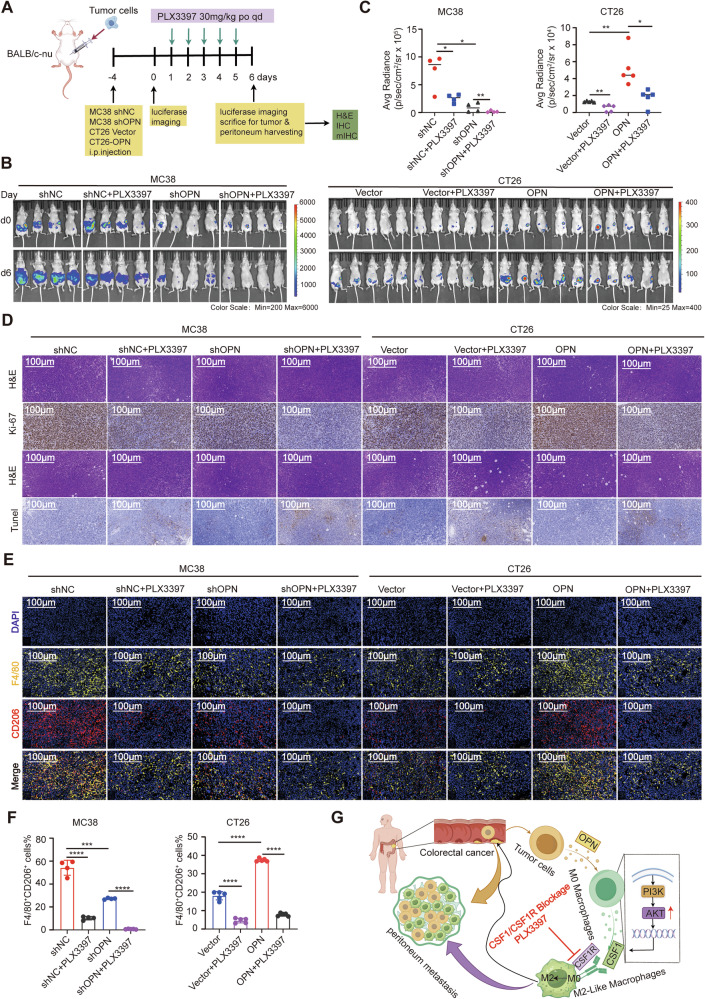
CSF1R inhibitor abrogates M2-TAMs-mediated CRC metastasis in vivo. **A** A schematic diagram of the in vivo mouse peritoneal metastasis experiment. BALB/c-nu nude mice were intraperitoneally injected with 5 × 10^5^ MC38sh (*n* = 4) or CT26-OPN cells (*n* = 5). Peritoneal metastasis was assessed via IVIS 4 days after injection. Mice were grouped based on bioluminescence values and orally treated with 30 mg/kg PLX3397 or control solvent for 5 days. Post-treatment, metastasis was re-evaluated via IVIS, and tumors were collected for analysis (e.g., **D**–**F**). **B** Bioluminescence imaging of peritoneal metastatic tumors pre- and post-PLX3397 treatment. **C** Quantitative analysis of bioluminescence imaging post-treatment. **D** Representative staining of tumor sections: H&E (top), Ki-67 (middle), and TUNEL (bottom). Scale bar: 100 μm. **E** mIHC analysis of M2-TAMs (F4/80^+^CD206^+^) in tumors. **F** Proportion of M2-TAMs (F4/80^+^CD206^+^) in each group. **G** Mechanistic insights are summarized: Tumor-derived OPN activates PI3K/AKT signaling in macrophages, inducing CSF1 secretion and polarization of macrophages to the M2 phenotype dependent on CSF1R. This crosstalk facilitates reciprocal migration between CRC cells and TAMs, promoting metastatic progression. PLX3397 disrupts this loop by blocking CSF1R, thereby reducing M2 polarization and CRC tumor metastasis. IVIS, in vivo imaging system. All data are shown as mean ± SD; *p < 0.05; **p < 0.01; ***p < 0.001; ****p < 0.0001; ns, not significant.

To further investigate whether inhibiting the CSF1/CSF1R axis can reduce M2-TAMs infiltration and tumor metastasis, we established CRC peritoneal metastasis models in nude mice using MC38shNC and MC38shOPN, as well as CT26 Vector and CT26-OPN cells. Once peritoneal metastatic tumor signals were visible by live imaging, mice were treated with either the vehicle control or the CSF1R inhibitor PLX3397 until the end of the study (Fig. 7A). The results showed that the OPN^high^ groups (MC38shNC and CT26-OPN) had more metastatic tumors than the OPN^low^ groups (MC38shOPN and CT26 Vector). Furthermore, treatment with PLX3397 significantly inhibited tumor metastasis compared to the control group (Fig. 7B, C). IHC staining of metastatic tumors revealed that PLX3397 treatment suppressed tumor proliferation, as indicated by decreased Ki-67 staining, and promoted apoptosis, as shown by increased TUNEL staining (Fig. 7D). Correspondingly, ELISA analysis demonstrated a decrease in CSF1 concentration within mouse serum following treatment (Fig. S7A). To further validate that inhibiting the CSF1/CSF1R axis reduces M2-TAMs infiltration in metastatic foci, mIHC analysis demonstrated that PLX3397 treatment selectively depleted M2-TAMs infiltration in tumors (Fig. 7E, F). However, IHC analysis revealed no significant effect on M1 macrophage infiltration (Fig. S7B, C).

Based on these findings, we present the model illustrated in Fig. 7G to explain the underlying mechanisms. CRC-derived OPN activates the PI3K/AKT signaling pathway in macrophages, leading to CSF1 secretion. The CSF1/CSF1R pathway then polarizes macrophages into an M2-TAMs phenotype, facilitating mutual chemotactic migration of M2-TAMs and CRC cells. Furthermore, blocking the CSF1/CSF1R pathway reduces both M2-TAMs chemotaxis and CRC metastasis.

## Discussion

Metastasis is a major challenge in the clinical treatment of CRC, underscoring the urgent need to unravel molecular drivers and identify actionable therapeutic targets. Our study establishes OPN as a pivotal mediator of CRC metastasis through macrophage-dependent mechanisms. We demonstrate that CRC tumor-derived OPN plays a critical role in promoting macrophage recruitment and M2 polarization via the PI3K/AKT/CSF1/CSF1R signaling axis. This process fosters a pro-metastatic microenvironment (Fig. 7G). Critically, blocking CSF1R with PLX3397 attenuates TAMs recruitment and M2 polarization, thereby suppressing CRC metastasis. These findings position OPN as a valuable biomarker to identify patients who may benefit from macrophage-targeted therapies, and CSF1R as a potential therapeutic target for advanced CRC.

Previous studies have reported that high levels of OPN in CRC enhance cancer-cell invasion and metastasis [31, 32]. In contrast, our study found that neither OPN knockdown nor overexpression significantly altered CRC cell invasion or metastasis, suggesting that OPN’s contribution to metastasis is microenvironment-dependent rather than cell-autonomous. Previous studies have demonstrated that cell-autonomous addiction, wherein cancer cells rely on intrinsic signaling pathways for survival and proliferation, is context-specific. For instance, in breast or intra-hepatic cholangiocarcinoma, OPN directly activates β-catenin, PI3K/AKT, or IRF1–HOTAIR cascades within the cancer cell. In contrast, CRISPR/Cas9 deletion of *SPP1* in HCT-116 or SW620 CRC cells only marginally impairs proliferation, migration or liver-colonization ability [42]. Additionally, the metastatic phenotype appears to be driven primarily by host-derived, rather than tumor-derived OPN. Elegant murine experiments show that *SPP1*^−/−^ hosts resist liver metastasis despite engraftment of OPN^-high^ MC-38 CRC cells; whereas *SPP1*^+/+^ hosts permit dissemination even when the injected tumor cells are *SPP1*^−/−^ [43]. Collectively, these findings indicate that OPN drives CRC metastasis primarily through paracrine-mediated remodeling of the hepatic and bone microenvironments rather than through cell-intrinsic signaling. Our observation that neither overexpression nor knockdown influenced CRC invasion, therefore aligns with emerging evidence that OPN’s contribution to metastasis is niche-dependent, not cancer cell-autonomous. Previous studies have shown a strong correlation between OPN and macrophages [22, 29, 44]. Single-cell analyses have illuminated macrophage-tumor crosstalk via OPN-CD44 [45] and fibroblast-macrophage interactions mediated by *SPP1* (OPN) in CRC [22], aligning with our findings that OPN orchestrates reciprocal chemotaxis-mutual attraction between CRC cells and M2-TAMs. OPN has been shown to regulate immune cell function and immune evasion, and its expression is associated with the infiltration of various immune cells, such as M2-TAMs [29, 30]. M2-TAMs have also been demonstrated to contribute to CRC progression [46, 47]. Recent studies have reported that tumor-derived OPN promotes macrophage chemotactic migration and M2-like polarization, thereby facilitating tumor progression and metastasis [33–35]. These results collectively posit OPN as a stromal-immune modulator rather than a standalone oncogenic driver.

However, the relationship between CRC-derived OPN and M2-like macrophages, as well as its clinical significance, remains poorly understood. Our mechanistic dissection identifies CSF1/CSF1R signaling as the linchpin of OPN-mediated macrophage reprogramming. RNA-seq profiling and functional validation revealed that CRC-derived OPN activates PI3K/AKT signaling in macrophages, upregulating CSF1 secretion to amplify an autocrine CSF1/CSF1R loop. This feedforward circuit sustains the TAMs recruitment, M2 polarization, and pro-metastatic niche formation. Notably, hyperactivation of the PI3K/AKT pathway by OPN mirrors observations in bladder [48] and esophageal cancers [35], suggesting a conserved mechanism across malignancies. The spatial restriction of CSF1R expression to TAMs in CRC tissues [49] further underscores the therapeutic rationale for CSF1R inhibition to disrupt this axis. The efficacy of PLX3397 in curbing OPN-driven metastasis in preclinical models highlights the translational potential of CSF1R blockade. Preclinical evidence supports CSF1R inhibition as a strategy to reprogram immunosuppressive TAMs, enhance cytotoxic T-cell infiltration, and potentiate checkpoint immunotherapy [50–52]. Our data extend these observations to CRC, demonstrating that PLX3397 abrogates OPN-high tumor metastasis by dismantling macrophage-dependent support networks. Tumor cell-derived OPN has been identified as a novel immune checkpoint that suppresses T-cell activation and confers systemic tumor immune tolerance [53]. Supporting its therapeutic relevance, a clinical study on bone metastasis revealed that blocking OPN enhances responses of extraosseous tumors to immune-checkpoint blockade (ICB) in patients with skeletal lesions [54]. These findings highlight OPN as a promising immunomodulatory target. Consequently, emerging combinatorial approaches such as coupling CSF1R inhibitors with BRAF-targeted therapy, PD-1/PD-L1 blockade [55] or OPN blockade, warrant further exploration in CRC to overcome stromal-mediated immunotherapy resistance.

Despite the significant role of OPN in CRC progression, this study has certain limitations. First, although we explored CRC tumor-derived OPN in activating the PI3K/AKT/CSF1-CSF1R signaling axis, the receptor through which OPN activates this pathway remains undefined. OPN contains a canonical arginine-glycine-aspartic acid (RGD) motif that interacts with multiple integrins and CD44, thereby regulating proliferation, adhesion, invasion, migration, and fibrosis [36, 56, 57]. In esophageal carcinoma, OPN has been reported to drive M2-like TAMs polarization via the CD44/PI3K/AKT cascade; in liver diseases, OPN activates CD44 and integrins in an autocrine/paracrine manner to trigger PI3K/AKT and MAPK signaling [36]. Future work will therefore elucidate the precise mechanism and the cognate receptor by which OPN will engage the PI3K/AKT/CSF1-CSF1R pathway. Second, the downstream effectors linking PI3K/AKT to CSF1 secretion remain undefined. We also need to further explore the effects of combining PLX3397 treatment with other drugs in vivo. Finally, prospective studies are needed to validate and confirm the clinical applicability and utility of OPN as a CRC biomarker and the use of PLX3397.

In conclusion, our findings indicate that OPN derived from CRC tumor cells plays a critical role in promoting macrophage recruitment and M2-TAMs polarization, mediated by the PI3K/AKT/CSF1-CSF1R axis. Additionally, OPN-driven M2-TAMs infiltration is crucial for CRC tumor growth and metastasis. Finally, blocking CSF1R inhibits TAMs recruitment, M2 polarization, and CRC metastasis. These findings highlight that OPN serves as a valuable biomarker for guiding macrophage-targeted strategies, while CSF1R emerges as a potential therapeutic target for advanced CRC.

## Materials and methods

### Cell cultures

DLD1 (RRID: CVCL_0248), SW480 (RRID: CVCL_0546), THP-1 (RRID: CVCL_0006), HCT8 (RRID: CVCL_2478) and CT26 (RRID: CVCL_7254) cells were purchased from the American Type Culture Collection (ATCC), and MC-38 (RRID: CVCL_B288) was purchased from the National Infrastructure of Cell Line Resource (Beijing, China). All cells were cultured in ATCC-recommended media (Gibco) supplemented with 10% fetal bovine serum (FBS) and 1% antibiotics (penicillin and streptomycin) at 37 °C with 5% CO_2_. We confirmed that all cells were free of contamination by comparing their genomic profiles with the ATCC short tandem repeat (STR) database and detecting mycoplasma-specific sequences in the supernatant using standard PCR-based procedures. All cells were tested for mycoplasma contamination every two months, and all tests were negative.

To generate CM, human CRC cell lines (SW480, DLD1, HCT8) were seeded in 10 cm dishes and allowed to reach 90% confluence. The medium was then replaced with DMEM supplemented with 1% penicillin and streptomycin (pen/strep) and 10% FBS, and the cells were incubated for 48 hours. The medium was collected, filtered through a 0.8 µm filter to remove cell debris, and stored at −80 °C. For collecting CM from M2-like macrophages, THP-1 cells were treated with PMA (10 ng/ml, Sigma Aldrich, Cat#:P8139) for 24 hours, followed by incubation with IL-4 (25 ng/ml, Sino Biological, Cat#:GMP-11846-HNAE)and IL-13 (25 ng/ml, Sino Biological, Cat#:10369-HNAC) for 48 hours to generate THP-1-derived M2-like macrophages. The medium was then replaced with RPMI 1640 supplemented with 1% pen/strep and 10% FBS, and the cells were incubated for 24 hours. The medium was collected, filtered through a 0.8 µm filter, and stored at −80 °C.

### Co-culture assay

A total of 5 × 10^5^ SW480-shNC, SW480-shOPN, HCT8-Vector, and HCT8-OPN cells were seeded in the upper chamber of a six-well transwell plate with 0.4 µm porous polycarbonate membranes (LABSELECT 14112, Beijing, China). PMA-treated THP-1 cells (1 × 10^6^) were added to the lower chamber. After 24 hours of separate culture of each cell type, the cells were co-cultured for 48 hours, then, macrophages and supernatants were collected for analysis. Experiments were performed three times.

### Western blotting

Proteins were extracted from cells using RIPA buffer (Fujian Herui Biological Technology Co., Ltd, Cat#:HRX0087). Protein concentration was measured using a BCA protein assay kit (Fujian Herui Biological Technology Co., Ltd, Cat#:HRX0121). Equal amounts of protein were separated by 12% SDS-PAGE and transferred to PVDF membranes (Merck Millipore, Boston, MA, USA). Membranes were blocked with 5% skim milk in TBST for 1 hour and then incubated with primary antibodies against human and mouse OPN (Santa Cruz Biotechnology, Cat#:sc-21742, RRID:AB_2194997, diluted at 1:200), anti-PI3K (Zenbio, Wuhan, China, Cat#:R22768, RRID:AB_2863407, diluted at 1:200), anti-phospho-PI3K (Zenbio, Wuhan, China, Cat#:341468, RRID:AB_3675929, diluted at 1:200), anti-AKT (Zenbio, Wuhan, China, Cat#:342529, RRID:AB_3675962, diluted at 1:200), anti-phospho-AKT (Zenbio, Wuhan, China, Cat#:381555, RRID:AB_3675963, diluted at 1:200), anti-FLAG (Sigma Aldrich, Cat#:F1804, RRID:AB_262044, diluted at 1:1000), anti-CSF1R (Abcam, Cat#:ab229188, RRID:AB_2894766, diluted at 1:500) and anti-GAPDH (Proteintech, Cat#:60004-1-lg, RRID:AB_2721282, diluted at 1:3000). The secondary antibodies used were anti-mouse (Proteintech, Cat#:SA00001-1, RRID:AB_2722565, diluted at 1:3000) or anti-rabbit IgG-HRP (Proteintech, Cat#:SA00001-2, RRID:AB_2722564, diluted at 1:3000). Protein band density was analyzed using ImageJ software (RRID:SCR_003070). Experiments were performed three times.

### Multiplex fluorescent immunohistochemical staining (mIHC)

Formalin-fixed, paraffin-embedded tumor and adjacent tissue sections were stained according to the instructions of the four-color multiplex fluorescent immunohistochemical staining kit (Absin, Cat#:abs50012). Sections were blocked with goat serum (ZSGB-BIO, Cat#:ZLI-9056) before antibody incubation. The following primary antibodies were used: CK (Proteintech, Cat#:82428-1-RR, RRID:AB_3086478, diluted at 1:1000), OPN (SANTA, Cat#:sc-21742, RRID:AB_2194997, diluted at 1:25), F4/80 (Cell Signaling Technology, Cat#:70076, RRID:AB_2799771, diluted at 1:500), and CD206 (Cell Signaling Technology, Cat#:24595, RRID:AB_2892682, diluted at 1:400). Nuclei were stained with DAPI (diluted at 1:100) prior to mounting. All sections were scanned using a fluorescence scanner (TissueFAXS Plus-S, TissueGnostics).

### RNA sequencing

After co-culturing SW480-shOPN#2 and SW480-shNC cell CM with PMA-treated THP-1 cells for 48 hours, total RNA was extracted from THP-1-derived macrophages using Trizol reagent. Three independent experimental replicates and three technical replicates per sample were sequenced. Deep RNA sequencing (RNA-seq) was performed on the illumina Novaseq™ X Plus platform (RRID:SCR_024568) (LC-Bio Technology CO., Ltd. Hangzhou, China), and the sequencing data were analyzed using tools available on the LC-Bio Technology website (https://www.omicstudio.cn/home). Differential gene expression analysis was conducted, and heatmaps were generated using Z-score normalization (RRID:SCR_016418). Genes with a fold change (FC) ratio of FPKM (|*log2FC* | > 1.0, *p* < 0.05) were considered significantly differentially expressed. Kyoto Encyclopedia of Genes and Genomes (KEGG) (RRID:SCR_012773) pathway analysis was used to predict potential pathways through which OPN promotes macrophage polarization and migration. The RNA-seq data have been deposited in the NCBI Gene Expression Omnibus database (http://www.ncbi.nlm.nih.gov/geo/) under accession number GSE290045.

### Flow cytometry

Designated cells were collected and stained with the cell surface marker CD86 on ice for 30 minutes, followed by fixation/permeabilization using a fixation/permeabilization kit (eBioscience). After staining with CD206 and CD163 antibodies were added and incubated at room temperature in the dark for 30 minutes. Cell pellets were resuspended in PBS containing 2% FBS for flow cytometry analysis. All labeled cells were detected using a Beckman flow cytometry system and analyzed with CytExpert. The following fluorescent dye-labeled antibodies were used in this study: anti-Hu CD86 (FITC) (BioLegend, Cat#:374203, RRID:AB_2721573), anti-Hu CD163 (APC) (BioLegend, Cat#:333610, RRID:AB_2074533), and anti-Hu CD206 (PE) (InvivoGen, Cat#: 2647760, RRID:AB_2538347).

### Animal models and drug treatments

All immunodeficient BALB/c-nu mice (RRID:IMSR_CRL:490) (3–5 weeks old, female) were purchased from Guangdong GemPharmatech Co., Ltd (Guangdong, China) and housed under specific pathogen-free conditions in the animal facility of the Sixth Affiliated Hospital of Sun Yat-sen University. All animal research adhered to the ARRIVE guidelines and was carried out in accordance with protocols approved by the Institutional Laboratory Animal Care and Use Committee of The Sixth Affiliated Hospital, Sun Yat-sen University, China (IACUC-2024051301, IACUC-2024101803). The minimum number of animals necessary to achieve adequate statistical power was used, as required by the ARRIVE guidelines.

### Statistical analysis

All statistical analyses were performed using GraphPad Prism 9.0 software (RRID:SCR_002798) (GraphPadSoftware, San Diego, CA, USA). Data normality was assessed using the Shapiro-Wilk normality test. Data are presented as mean ± standard deviation (SD), as indicated. Differences between the two groups were compared using Student’s unpaired two-tailed *t* test in at least three independent experiments. Differences among multiple groups were evaluated using one-way analysis of variance (ANOVA) followed by Tukey’s post hoc test, based on at least three independent experiments. A *p* value < 0.05 was considered statistically significant. *p < 0.05; **p < 0.01; *** *p* < 0.001; ****p < 0.0001; ns not significant).

## Supplementary information

Manuscript-supplementary-Revision clean version

Original CD86 Flow cytometry Figure-S3A

Original WB Figure-3A

Original WB Figure-4C

Original WB Figure-6G

Original WB Figure-6H

Original WB Figure-S5A–C

Original WB Figure-S5F

## Data Availability

All data supporting the findings of this study are available with the article or from the corresponding author upon reasonable request.

## Code availability

The codes used in this paper are available from the lead contact upon request.
