# Supplementary material for: Colorectal cancer-derived osteopontin rewires macrophages into a pro-metastatic M2 state via the PI3K/AKT/CSF1-CSF1R axis
Source: Cell Death Discov. 2026 Feb 5;12:92. doi: 10.1038/s41420-026-02945-y (PMC12894849; doi:10.1038/s41420-026-02945-y)
Supplement: Supplementary file 1 — Manuscript-supplementary-Revision clean version [file 41420_2026_2945_MOESM1_ESM.docx]

**SUPPLEMENTARY MATERIALS AND METHODS**

**Study design**

The goal of this study is to investigate the impact of colorectal cancer-derived OPN on the mutual chemotaxis between OPN and M2-TAMs in promoting tumor metastasis. Additionally, we aim to explore the underlying molecular mechanisms and to evaluate the therapeutic efficacy of CSF1R inhibitors. We assessed OPN expression in CRC tumors by analyzing public databases, examining clinical tumor samples, and performing multiplex immunofluorescence (mIHC). Transgenic cell line models, flow cytometry, qPCR, Western blotting, transcriptomic analysis, macrophage co-cultures, transwell assays, ELISA, and Human Cytokine Antibody Array were employed to evaluate the mutual chemotaxis between CRC-derived OPN and M2-TAMs and to investigate their molecular mechanisms. Furthermore, the impact of blocking the CSF1/CSF1R axis on the M2-like polarization of TAMs and tumor metastasis was assessed using *in vitro* macrophage co-cultures, TUNEL assay, mIHC, animal metastasis models, and *in vivo* drug studies.

**Human samples**

Under strict adherence to the ethical guidelines authorized by the Institutional Review Board of Sun Yat-sen University (2024ZSLYEC-638), primary tumor tissues, adjacent normal tissues, and metastatic tissues were collected from colorectal cancer (CRC) patients who underwent surgery between January 2016 and December 2023. Additionally, peripheral blood samples were obtained from healthy individuals and stage II-IV CRC patients from the pathology tissue bank of the Sixth Affiliated Hospital of Sun Yat-sen University. None of the CRC patients received any preoperative cancer treatment.

RNA sequencing was performed on tissue microarrays constructed from 6 pairs of matched colorectal cancer and adjacent normal tissues. Differential expression analysis of osteopontin was then conducted using the limma package in R. Following this, qRT-PCR analysis was employed to assess differences in OPN mRNA levels in an independent cohort comprising 23 pairs of matched CRC and adjacent normal tissues. Finally, enzyme-linked immunosorbent assay (ELISA) was performed on peripheral blood samples from healthy controls and patients with stage II-IV CRC to evaluate differences in OPN protein levels.

**GEO and TCGA databases**

The DESeq2 (RRID:SCR_000154) tool was used to the CRC-related GSE142279 dataset obtained from the Gene Expression Omnibus (GEO) (RRID:SCR_005012) database ([https://www.ncbi.nlm.nih.gov/](https://www.ncbi.nlm.nih.gov/)). Additionally, the UALCAN (RRID:SCR_015827) online database ([https://ualcan.path.uab.edu/analysis.html](https://ualcan.path.uab.edu/analysis.html)) was used to analyze the expression levels of OPN and the prognosis of patients in CRC-related datasets from TCGA.

**Plasmids**

Lentiviral plasmids containing full-length human OPN (NM_001040060.2) and mouse OPN (NM_001204201.1) were generated using the recombinant vector pLVX-3Flag-MCS-IRES-Puro (RRID:Addgene_140240). The empty vector control plasmid was purchased from Guangzhou IGE Biotechnology Co., Ltd. (Guangzhou, Guangdong, China). The specific shRNA sequences used in this study were: human HushOPN#1: 5′-AGTTGAATGGTGCATACAAGG-3′; HushOPN#2: 5′-CGAGGAGTTGAATGGTGCATA-3′. Human OPN gene silencing was performed by lentiviral transduction using pLKO.1-CMV-copGFP-PURO (RRID:Addgene_139470), and the corresponding negative control was obtained from Beijing Tsingke Biotech Co., Ltd. (China). For mouse OPN gene silencing, the shOPN sequence was: 5′-GGATGTGATCGATAGTCAAGC-3′. Gene silencing was performed using the lentiviral plasmid pLKO.1-U6-EF1a-copGFP-T2A-puro (RRID:Addgene_110318), and the corresponding negative control was obtained from Guangzhou IGE Biotechnology Co., Ltd. (Guangzhou, Guangdong, China).

**Cell proliferation assay**

For the proliferation assay, equal numbers of shOPN, OPN-OE, and their respective control CRC cell lines (DLD1, SW480, HCT8; seeding density: 4 × 10^3^ cells/well) were seeded into 96-well plates containing 200 µl of medium supplemented with 1% pen/strep and 10% FBS. Cell confluence percentage was continuously measured for 1 day using the IncuCyte system. This experiment was independently repeated three times. For the half-maximal inhibitory concentration (IC_50_) determination of inhibitors, CRC cell lines were added to 96-well plates, and after cell attachment, various concentrations of inhibitors were added, and cells were cultured for 3 days. Cell viability was assessed using the Cell Counting Kit (CCK)-8 assay according to the manufacturer’s instructions. The IC_50_ and maximum non-toxic drug concentration were calculated using GraphPad Prism software. Experiments were performed three times.

**Wound healing assay**

shOPN, OPN-OE, and their respective control CRC cell lines (DLD1, SW480, HCT8; seeding density: 1 × 10^6^ cells/well) were seeded in a wound maker (Essen Bioscience). After the cells reached full confluence, the wound maker was removed, and the medium was replaced with 1 ml of fresh serum-free medium. The cells were then incubated at 37°C in the IncuCyte system, and wound width was measured every 2 hours. Images were captured after 24 hours. Experiments were performed three times.

**Transwell migration/invasion assay**

Transwell migration and invasion assays were performed using 24-well transwell inserts with 8 µm pores (Corning, NY, USA). shOPN, OPN-OE and their respective control cells (DLD1, SW480, HCT8), were suspended in serum-free medium and added to the upper chamber of each insert. For the invasion assay, inserts were coated with extracellular matrix (ECM; Corning, NY, USA) and 500 µl of medium containing 10% FBS was added to the lower chamber. Cells were cultured at 37°C for 24 hours. After incubation, cells were fixed with 4% paraformaldehyde and stained with 0.1% crystal violet. Images of the membranes were captured using an Olympus microscope (Tokyo, Japan), and the number of migrated cells was counted in five randomly selected fields. Experiments were performed three times.

To study the chemotactic migration of M2-TAMs toward CRC cells, CRC cells were added to the upper chamber, while the lower chamber contained 500 µl of fresh medium with 10% FBS mixed at a 1:1 ratio with M2-TAMs CM. After 24 hours of incubation at 37°C, cells were fixed with 4% paraformaldehyde and stained with 0.1% crystal violet. Images were captured using an Olympus microscope, and the number of migrated cells was counted in five randomly selected fields. Experiments were performed three times.

To study the role of OPN in recruiting macrophages, HCT8-OPN and DLD1-OPN cells were pretreated with 20 µM OPN inhibitor Compound 11 for 24 hours, followed by replacement with fresh medium containing 10% FBS. After another 24 hours, CM was collected. PMA-treated THP-1 cells were added to the upper chamber of 8 µm transwell inserts, and 500 µl of fresh medium containing 10% FBS mixed at a 1:1 ratio with CRC cell CM was added to the lower chamber. After 24 hours of incubation, cells were fixed with 4% paraformaldehyde and stained with 0.1% crystal violet. Images were captured using an Olympus microscope, and the number of migrated cells was counted in five randomly selected fields. Experiments were performed three times.

To assess the effect of CSF1R blockade on macrophage migration, PMA-treated THP-1 cells were added to the upper chamber of 8 µm transwell inserts, and 500 µl of fresh medium containing 10% FBS mixed at a 1:1 ratio with CM from SW480shNC, SW480shOPN, HCT8 Vector, HCT8-OPN cells was added to the lower chamber. The CSF1R inhibitor PLX3397 (10 µM) was added to the upper chamber containing SW480shNC and HCT8-OPN CM. After 24 hours of incubation at 37°C, cells were fixed with 4% paraformaldehyde and stained with 0.1% crystal violet. Images were captured using an Olympus microscope, and the number of migrated cells was counted in five randomly selected fields. Experiments were performed three times. The OPN inhibitor Compound 11 was purchased from MedChemExpress (New Jersey, USA), and the CSF1R inhibitor pexidartinib (PLX3397) was purchased from Selleck Chemicals (Houston, Texas, USA).

**qRT-PCR assays**

Total RNA was extracted using a total RNA extraction kit (GOONIE, Cat#:400-100). cDNA was synthesized using a cDNA reverse transcription kit (TOYOBO, Cat#:FSQ-301), and qPCR was performed in triplicate using 10 µl of SYBR master mix (Roche , Cat#:12239264001). Primer sequences are listed in the supplementary Table S1. Experiments were performed three times.

**Immunohistochemistry (IHC)**

Paraffin sections of CRC clinical samples, adjacent normal tissues, and mouse tumors were heated at 65°C for 2 hours, followed by two rounds of dewaxing in xylene substitute. Sections were sequentially immersed in 100%, 90%, and 70% ethanol and then in distilled water. Tris-EDTA antigen retrieval buffer (pH 9.0) (Servicebio) was used for heat-induced epitope retrieval in a microwave. Endogenous peroxidase activity was quenched by applying 3% H_2_O_2_ (Anji GaoKe) for 15 minutes, and sections were then blocked with goat serum (ZSGB-BIO, Cat#:ZLI-9056). Primary antibodies were diluted and applied to tissue sections, followed by overnight incubation at 4°C. After washing with PBS three times, goat anti-mouse/rabbit HRP-conjugated secondary antibodies (Servicebio) were applied. A DAB detection kit (Servicebio) was used for visualization. Nuclei were counterstained with hematoxylin (Servicebio). After dehydration by sequential immersion in 70%, 90%, and 100% ethanol and two rounds of immersion in xylene substitute, coverslips were mounted with neutral balsam (Servicebio). Sections were scanned using a slide scanning image system (Shenzhen, China). The antibodies used in this study were as follows: monoclonal antibody against mouse Ki-67 (Servicebio, Cat#:GB121141, RRID:AB_3083641, diluted at 1:600), Terminal deoxynucleotidyl transferase dUTP nick end labeling (TUNEL) kit (Servicebio, Cat#:G1507), anti-CD86 (Servicebio, Cat#:GB150054, RRID:AB_1544685, diluted at 1:600) and OPN (SANTA, Cat#:sc-21742, RRID:AB_2194997, diluted at 1:25). Staining intensity was graded as follows: no staining, score 0; weak staining, score 1; moderate staining, score 2; strong staining, score 3. The percentage of positively stained cells was scored as follows: 0-5%, score 0; 6-25%, score 1; 26-50%, score 2; 51-75%, score 3. The IHC score was obtained by multiplying the staining intensity score by the percentage score of positive cells.

**Human cytokine antibody array and ELISA**

CM from transfected SW480 and HCT8 cells or different concentrations of rhOPN were co-cultured with PMA-treated THP-1 cells for 48 hours. Subsequently, CSF1 levels in the co-culture supernatant were assessed using a human cytokine antibody array (AAH-CYT-3; RayBiotech®, Norcross, GA, USA) or a human M-CSF ELISA kit (MULTI SCIENCES, Cat#:EK1144) according to the manufacturer’s instructions. Additionally, OPN levels in the plasma of CRC patients and healthy individuals were assessed using a human OPN ELISA kit (MULTI SCIENCES, Cat#:EK1135). Mouse CSF-1 concentrations in serum were determined with the a mouse M-CSF ELISA kit (MULTI SCIENCES, Cat# :EK2144) according to the manufacturer's instructions.

**Animal models and drug treatments**

**Subcutaneous models**

A subcutaneous tumor model was used to evaluate the promoting role of OPN in vivo and its relationship with the M2-TAMs infiltration. Mice were randomly divided into four groups (*n*=5/group). MC38 shNC, MC38 shOPN, CT26 Vector, and CT26-OPN cells (5 × 10^5^ cells in 100 µL of phosphate buffer saline [PBS]) were injected subcutaneously into the left flank of nude mice. Tumor size was measured daily using calipers until the maximum tumor diameter exceeded 2 cm. Tumor volume was calculated using the formula: tumor volume (mm³) = (length × width²) × 0.5. After approximately 13 days, mice were euthanized, and subcutaneous tumors were harvested, weighed, photographed, fixed, and embedded in paraffin for H&E, IHC, and mIHC analysis.

**Transfer models and drug treatments**

To investigate the interaction between OPN and M2-TAMs in promoting metastasis *in vivo,* as well as the therapeutic effects of CSF1R inhibitors, we established a peritoneal metastasis model. Mice were randomly divided into four groups (MC38, *n*=8/group; CT26, *n*=10/group). MC38 shNC, MC38 shOPN, CT26 Vector, and CT26-OPN cells (5 × 10^5^ cells in 100 μL of phosphate-buffered saline [PBS]) were intraperitoneally injected into nude mice. Four days post-injection, mice bearing luciferase-expressing tumors were scanned using an in vivo imaging system (IVIS, Caliper Life Sciences). Within each group, mice were then randomly assigned to two subgroups based on fluorescence values: a control group and a treatment group. The treatment groups were received 30 mg/kg PLX3397 (Selleck; Houston, Texas, USA) orally once daily for 5 consecutive days, while the control groups received the corresponding solvent on the same schedule. After the final treatment, mice were scanned again using IVIS, euthanized, and peritoneal tumors were collected. The tumors were photographed, fixed, and embedded in paraffin for H&E staining, IHC, and multiplex immunohistochemical (mIHC) analysis.

**Randomization statement**

For the subcutaneous-tumor model, mice were stratified by body weight and then randomly allocated, ensuing that each group contained five animals.
For the peritoneal metastasis model, animals were first stratified based on the intraperitoneal fluorescence signal measured after tumor cell inoculation and evenly distributed: the MC38 cohort comprised four mice per group, and the CT26 cohort comprised five mice per group.

**Blinding**

Throughout the preclinical animal studies, investigators remained unaware of group allocation. After randomization, cages were labeled with numeric codes generated by an independent technician. These codes were not disclosed until data collection was complete. Drug administration and tumor size measurements were performed by an operator who had not participated in randomization or data analysis. In addition, for histological scoring, slides were digitized and assigned random numbers, and the image analyst remained blinded to treatment identity until statistical analysis was finalized.

**Supplementary Figures and Figure legends**

**
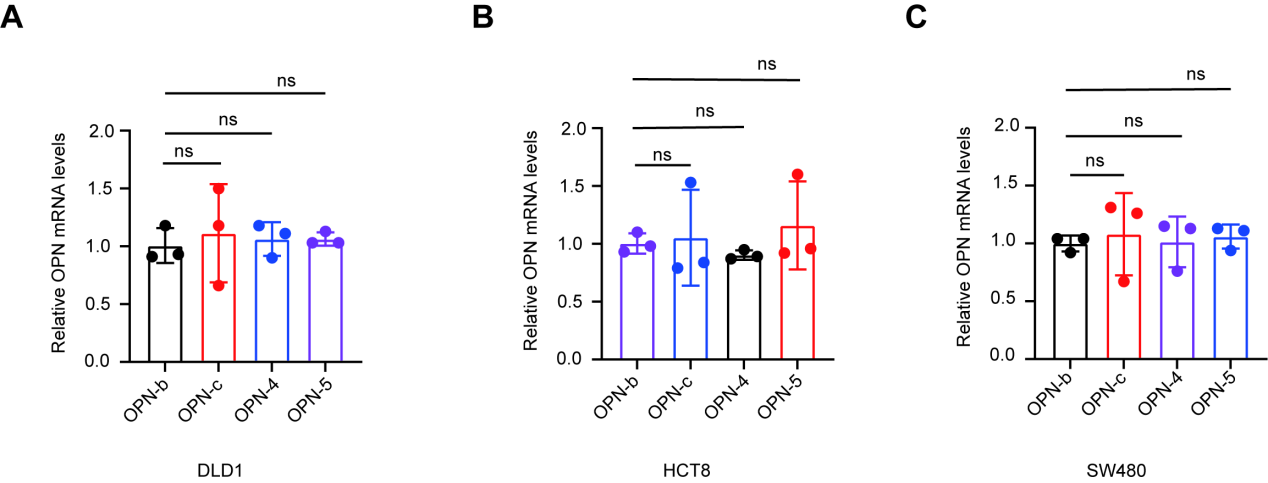
**

**Figure S1.** (**A-C**) The mRNA expression levels of OPN splice variants in CRC cells were quantified by qPCR. All data are shown as mean ± SD; ns indicates not significant.


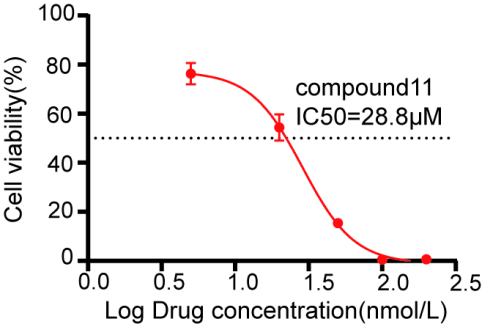


**Figure S2.** The half-maximal inhibitory concentration (IC_50_) value of the OPN inhibitor Compound 11 was evaluated using the CCK8 assay.


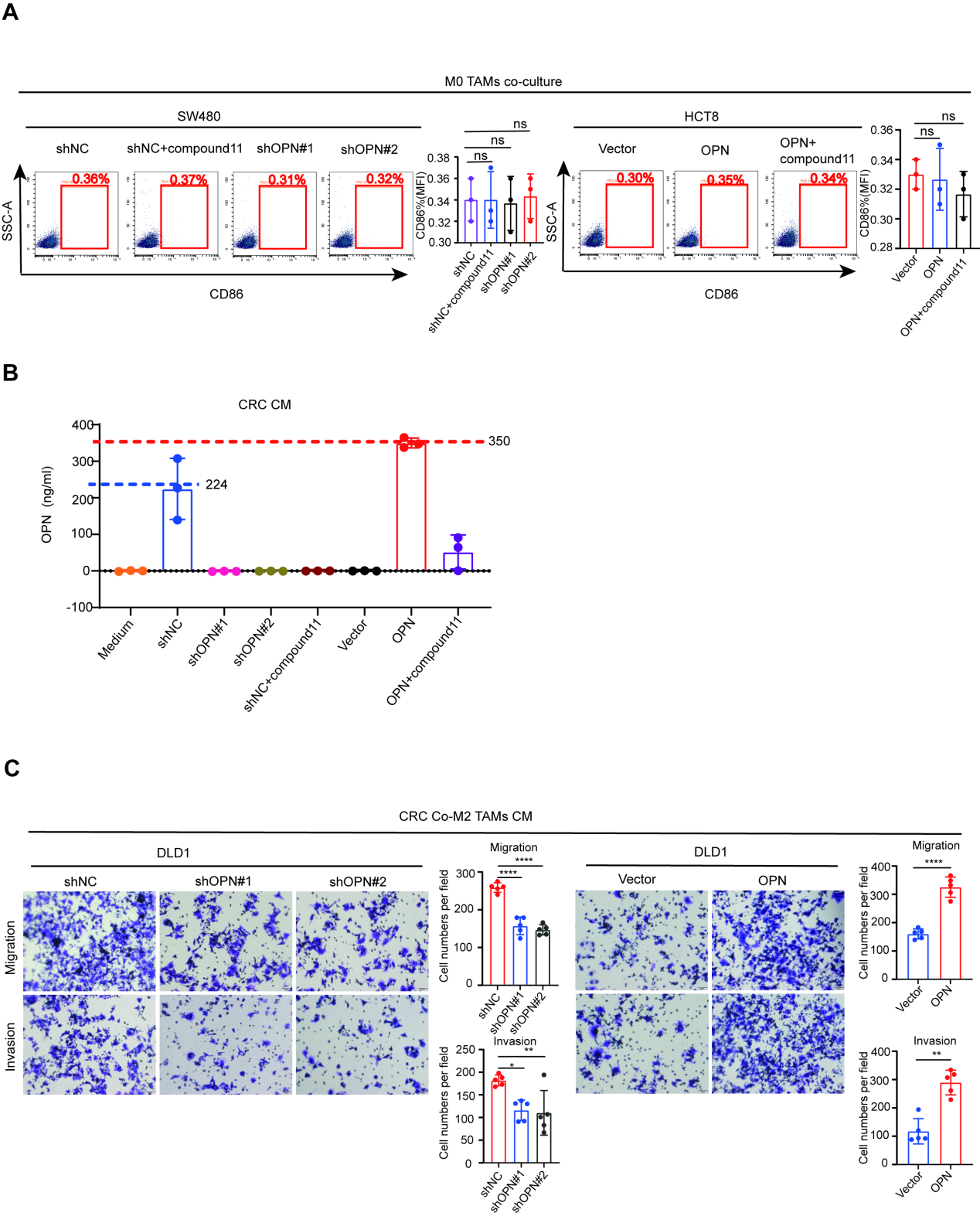


**Figure S3. OPN induces M2-like polarization of macrophages in CRC and promotes reciprocal migration.** (**A**) Flow cytometry analysis of CD86 expression on macrophages. (**B**) The concentration of OPN in CM, determined by ELISA, correlates with macrophage polarization. (**C**) Transwell migration and invasion assays of CRC cells cultured with CM from M2-TAMs are shown on the left, with migrated CRC cells counts quantified on the right. All data are shown as mean ± SD ; **p* < 0.05; ***p* < 0.01; ****p* < 0.0001.


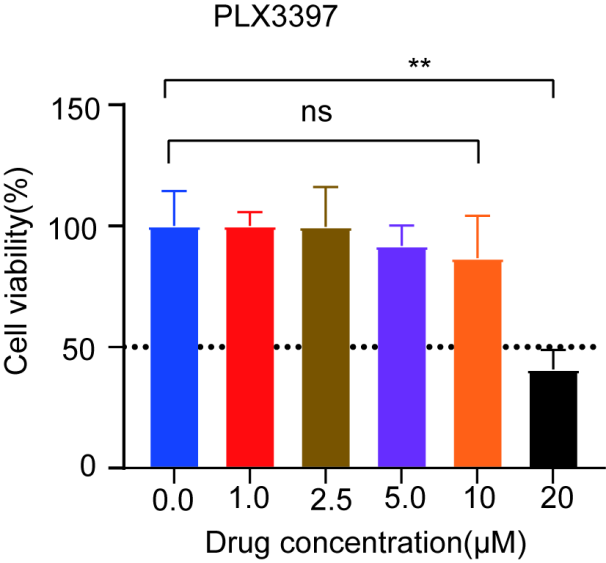


**Figure S4.** THP1 cell viability was assessed using the CCK8 assay following a 72-hour exposure to the indicated concentrations of the CSF1R inhibitor PLX3397. All data are shown as mean ± SD ; *n*=5, ***p* < 0.01.


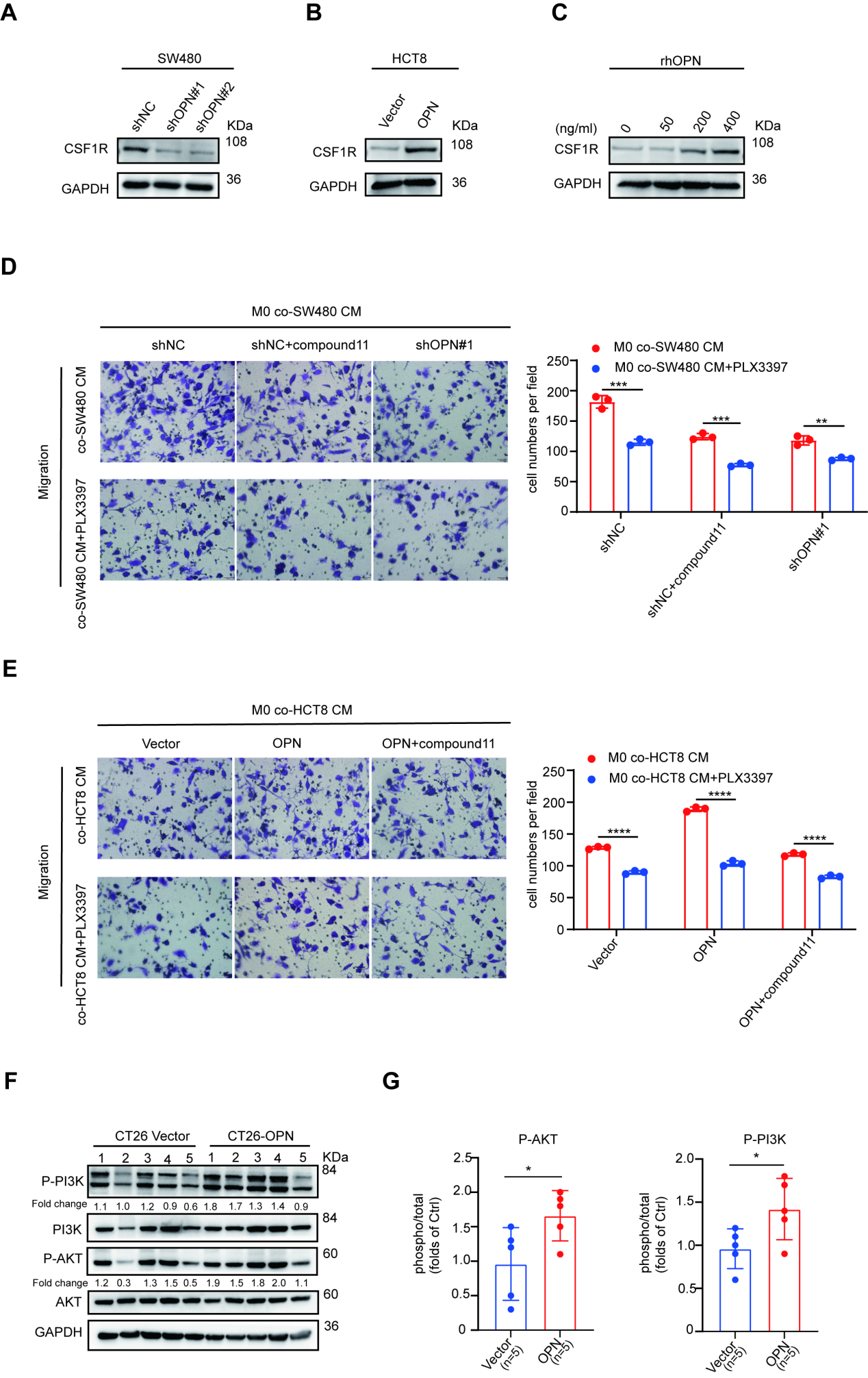


**Figure S5. OPN activates the PI3K/AKT signaling axis to promote macrophage secretion of CSF1.** (**A-B**) Macrophages treated with CRC cells’ CM for 72 hours showed CSF1R expression by Western blot. (**C**) Macrophages treated with rhOPN for 72 hours exhibited CSF1R expression by Western blot. (**D-E**) Transwell migration assays of PLX3397-treated macrophages co-cultured with compound 11-treated CRC cell CM (left), with quantitative results summarized (right). (**F**) Western blot analysis of phosphorylation of PI3K/AKT in CT26-Vector and CT26-OPN subcutaneous tumor xenografts (*n*=5/group). (**G**) Quantitative results of phospho-PI3K/AKT normalized to total PI3K/AKT proteins. All data are shown as mean ± SD; **p* < 0.05; ***p* < 0.01; ****p* < 0.001; *****p* < 0.0001.


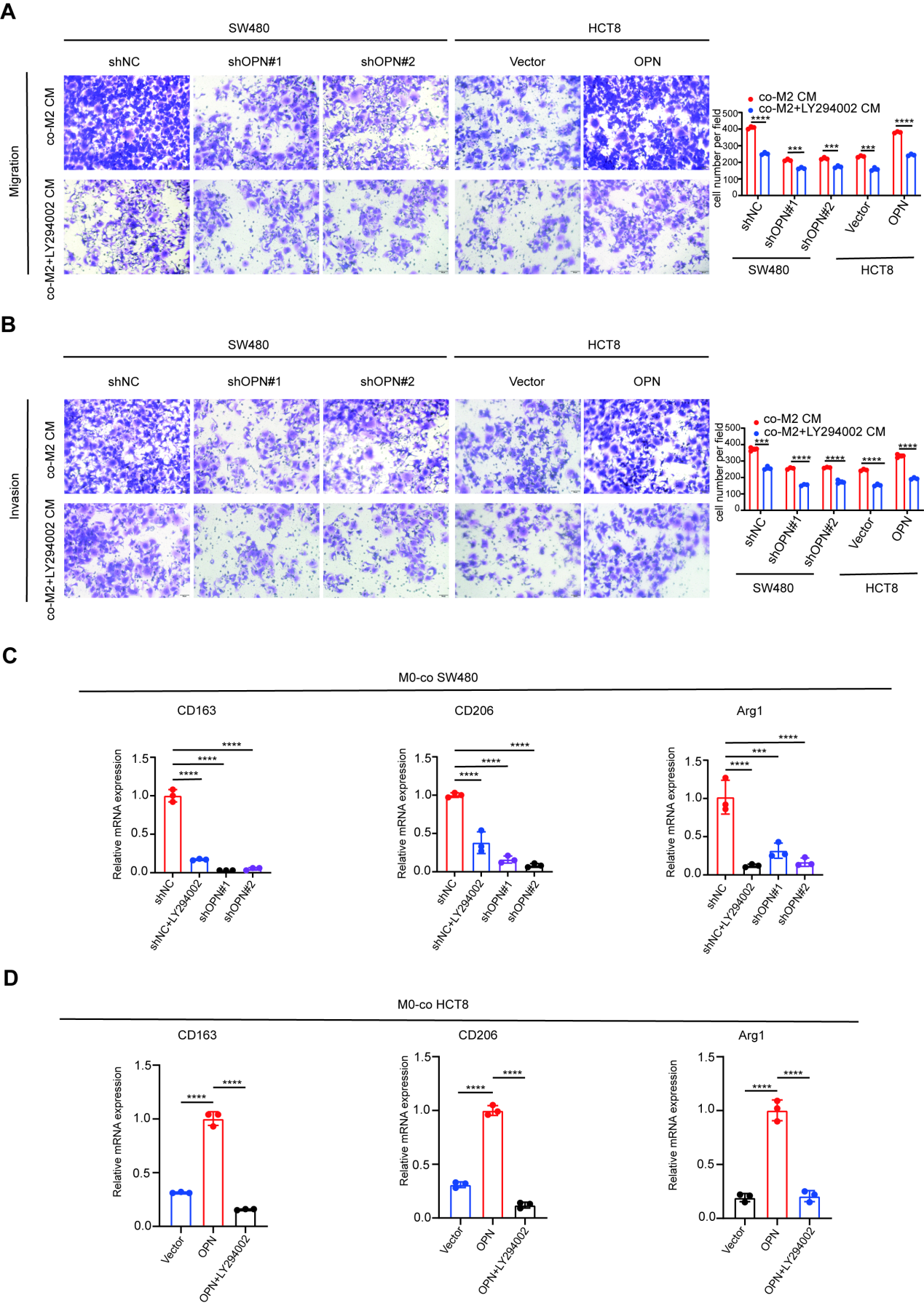


**Figure S6.** **The impact of blocking PI3K on CRC and macrophage polarization** . (**A-B**) Transwell migration and invasion assays of CRC cells (SW480 and HCT8) co-cultured with CM from M2-TAMs or CM from M2-TAMs treated with LY294002 (left), quantitative results are summarized (right). (**C-D**) Macrophages treated with CM from CRC cells or with LY294002-treated CRC cell CM for 72 hours showed M2 marker expression by qPCR. All data are shown as mean ± SD; ****p* < 0.001; *****p* < 0.0001.


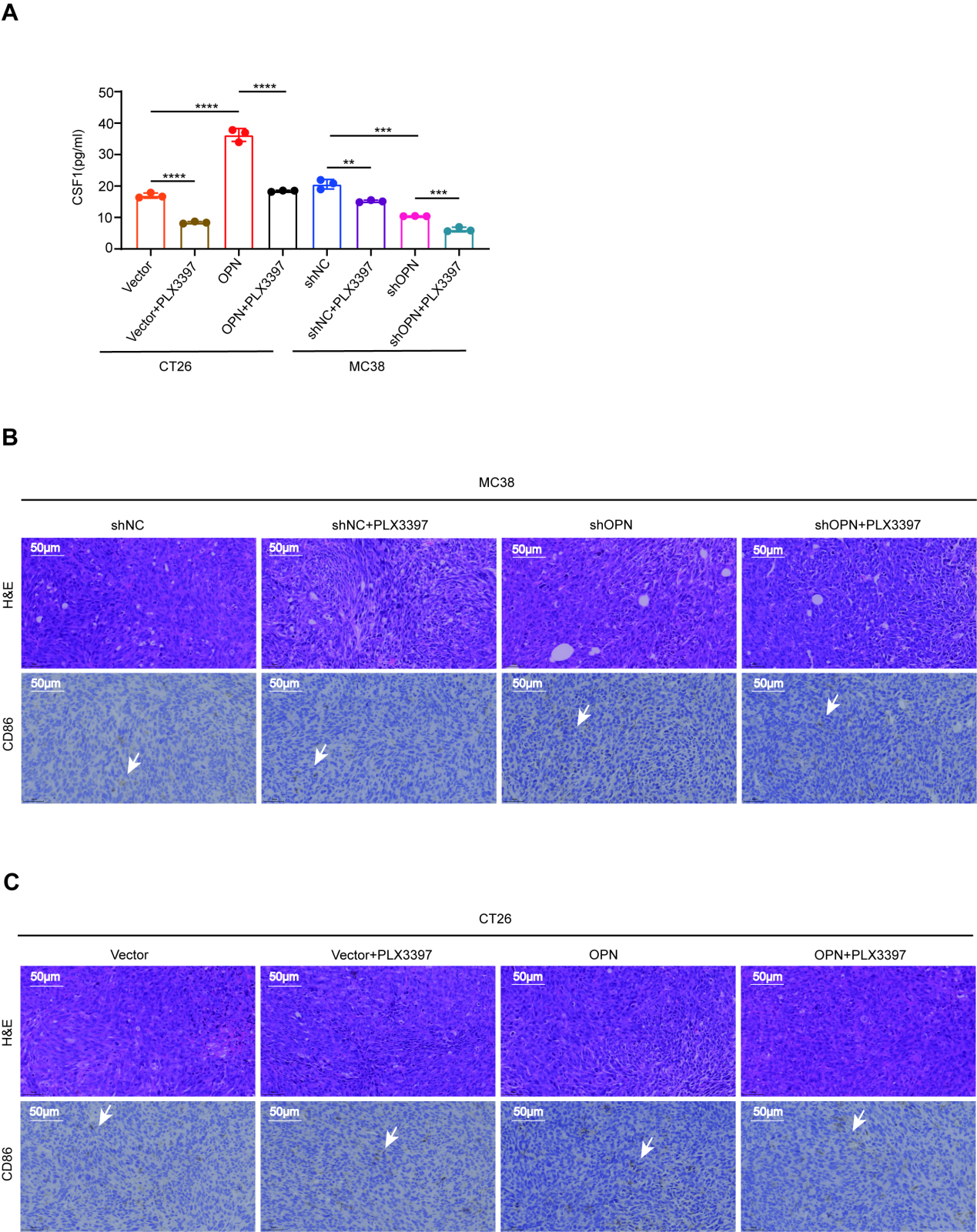


**Figure S7. CSF1R inhibitor abrogates M2-TAMs mediated CRC metastasis *in vivo*.** (**A**) The level of CSF1 in the serum of model mice by ELISA. (**B-C**) Representative staining of tumor sections: H&E (top) and CD86 (bottom). Scale bar: 50 μm. All data are shown as mean ± SD; ***p* < 0.01; ****p* < 0.001; *****p* < 0.0001.


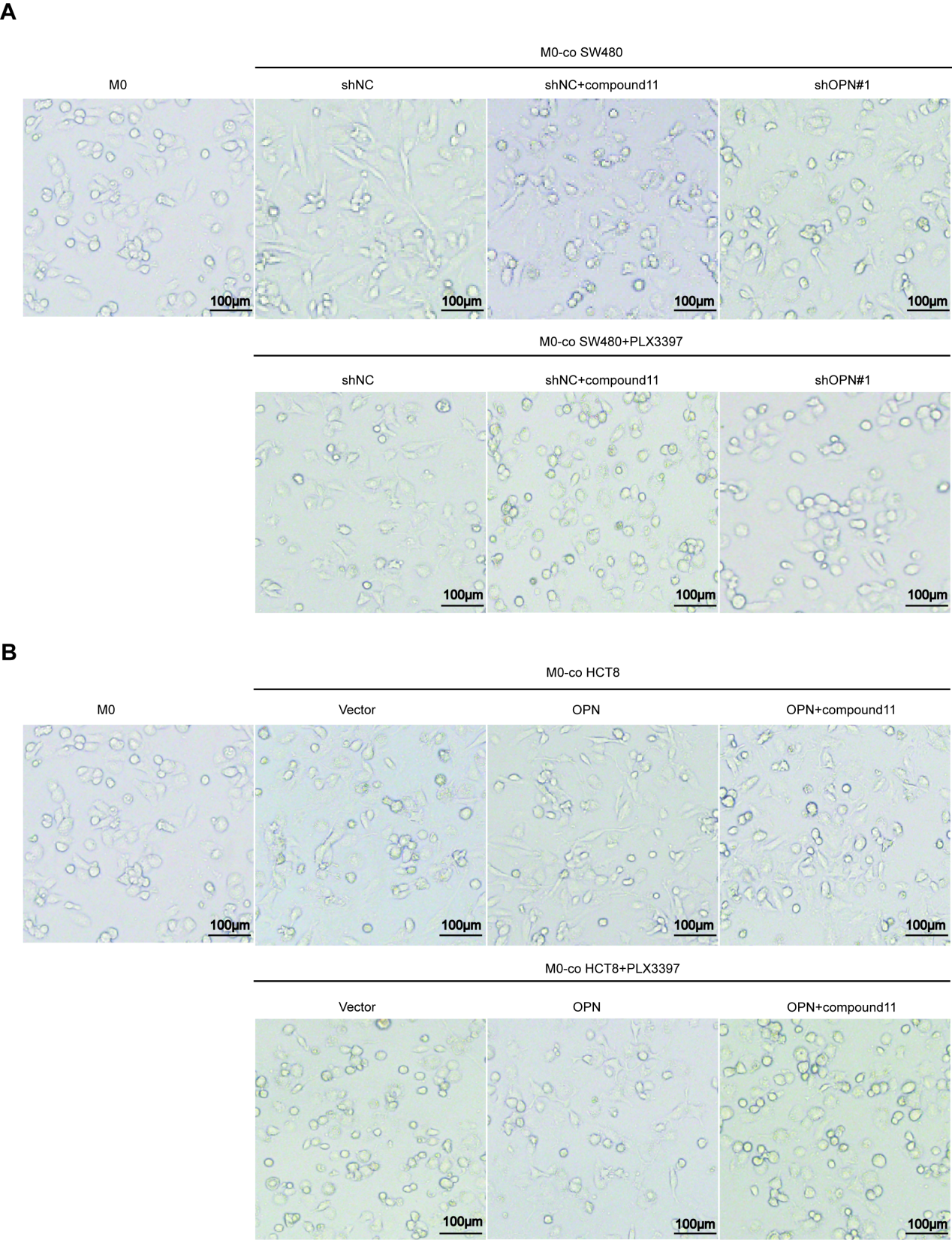


**Figure S8**. The image of polarized macrophage under all conditions and compare with PMA induced M0.

**Table S1.** Detailed information about resources

| Reagent | Source | Catalog no. |
| --- | --- | --- |
| Antibodies | | |
| OPN | Santa Cruz | sc-21742 |
| PI3K | Zenbio | R22768 |
| phospho-PI3K | Zenbio | 341468 |
| AKT | Zenbio | 342529 |
| phospho-AKT | Zenbio | 381555 |
| GAPDH | Proteintech | 60004-1-lg |
| Anti-Rabbit IgG | Proteintech | SA00001-2 |
| Anti-Mouse IgG | Proteintech | SA00001-1 |
| Ki-67 | Servicebio | GB121141 |
| F4/80 | Cell Signaling Technology | 70076 |
| CD206 | Cell Signaling Technology | 24595 |
| CK | Proteintech | 82428-1-RR |
| CD86 | BioLegend | 374203 |
| CD163 | BioLegend | 333610 |
| FLAG | Sigma Aldrich | F1804 |
| CSF1R | Abcam | Ab229188 |
| CD206 | InvivoGen | 2647760 |
| CD86 | Servicebio | GB150054 |
| Chemicals and fluorescent probes | | |
| Phorbol 12-myristate 13-acetate (PMA) | Sigma Aldrich | P8139 |
| Penicillin and streptomycin | Gibco | 15140122 |
| Basement membrane matrix | Corning | 354248 |
| Pexidartinib (PLX3397) | Selleck | S7818 |
| OPN inhibitor(compound11) | MedChemExpress | HY-146064 |
| Luciferin | Promega | P1041 |
| IL-4 | Sino Biological | GMP-11846-HNAE |
| IL-13 | Sino Biological | 10369-HNAC |
| LY294002 | Selleck | S1105 |
| Commercial kits | | |
| TUNEL kit | Servicebio | G1507 |
| M-CSF ELISA kit | MULTI SCIENCES | EK1144 |
| Mouse M-CSF ELISA kit | MULTI SCIENCES | EK2144 |
| OPN ELISA kit | MULTI SCIENCES | EK1135 |
| Total RNA Extraction Kit | GOONIE | 400-100 |
| RT Master Mix with gDNA Remover | TOYOBO | FSQ-301 |
| FastStart DNA Master SYBR Green I | Roche | 12239264001 |
| Cell Counting Kit-8(CCK-8) | APExBIO | K1018-500T |
| four-color multiplex fluorescent immunohistochemical staining kit | Absin | abs50012 |
| Human cytokine Array C3 kit | RayBiotec | AAH-CYT-3 |
| Recombinant proteins | | |
| OPN | Novoprotein | C544-50 |
| Oligonucleotides(RT-PCR) | | |
| Targeted Gene | Sequence | Source |
| Human GAPDH | For:GGAGCGAGATCCCTCCAAAAT  Rev:GGCTGTTGTCATACTTCTCATGG | IGE Biotechnology |
| Human SPP1(OPN5) | For:GAAGTTTCGCAGACCTGACAT  Rev:GTATGCACCATTCAACTCCTCG | IGE Biotechnology |
| Human CSF1R | For:GGGAATCCCAGTGATAGAGCC  Rev:TTGGAAGGTAGCGTTGTTGGT | IGE Biotechnology |
| Human CD163 | For:TTTGTCAACTTGAGTCCCTTCAC  Rev:TCCCGCTACACTTGTTTTCAC | IGE Biotechnology |
| Human CD206 | For:TCCGGGTGCTGTTCTCCTA  Rev:CCAGTCTGTTTTTGATGGCACT | IGE Biotechnology |
| Human Arg1 | For:GTGGAAACTTGCATGGACAAC  Rev:AATCCTGGCACATCGGGAATC | IGE Biotechnology |
| Human OPN4 | For:AAACAGGCTGATTCTGGAAGTTCTG  Rev:TGGAATTGAACTCTGTGTGCCTTTT | IGE Biotechnology |
| Human OPNc | For:AATTGCAGTGATTTGCTTTTGCCTC  Rev:CACAGCATTCTGCTTTTCCTCAGAA | IGE Biotechnology |
| Human OPNb | For:AGCAGCTTTACAACAAATACCCAGAT  Rev:TTGGACTTACTTGGAAGGGTCTGTG | IGE Biotechnology |
