# Supplementary figures and images for: Colorectal cancer-derived osteopontin rewires macrophages into a pro-metastatic M2 state via the PI3K/AKT/CSF1-CSF1R axis
Source: Cell Death Discov. 2026 Feb 5;12:92. doi: 10.1038/s41420-026-02945-y (PMC12894849; doi:10.1038/s41420-026-02945-y)

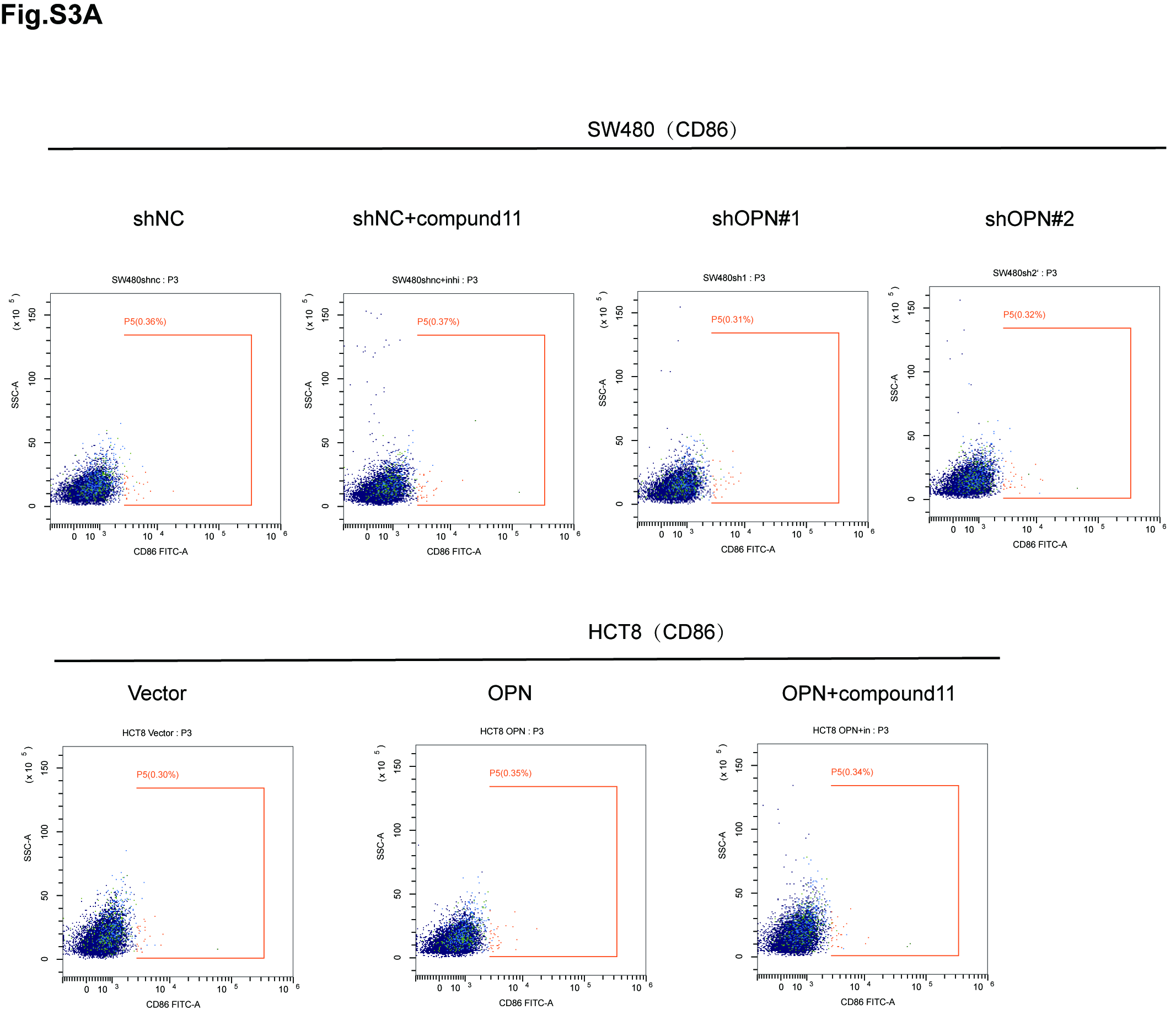

Supplement: Supplementary file 2 — Original CD86 Flow cytometry Figure-S3A [file 41420_2026_2945_MOESM2_ESM.tif]

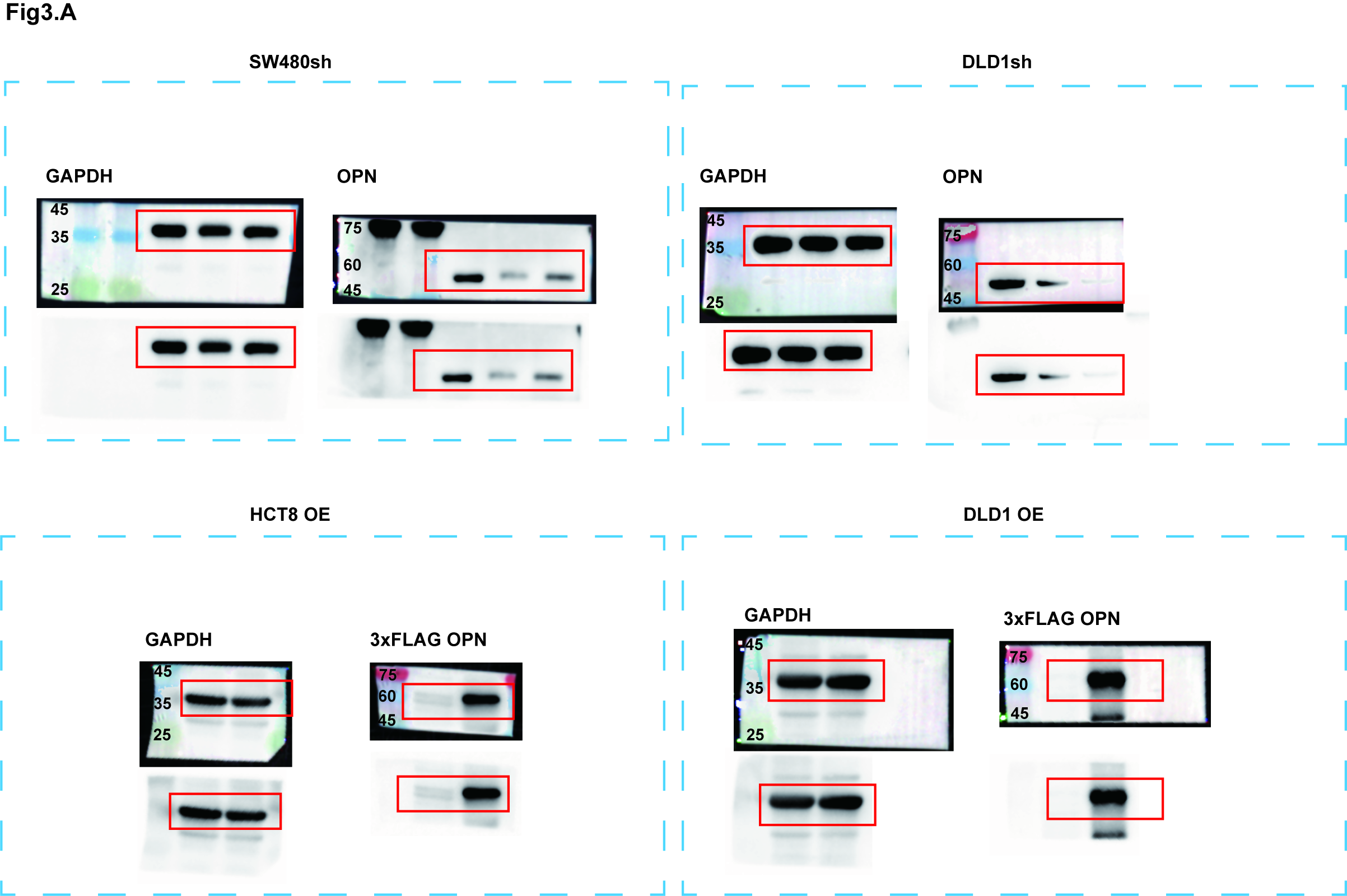

Supplement: Supplementary file 3 — Original WB Figure-3A [file 41420_2026_2945_MOESM3_ESM.tif]

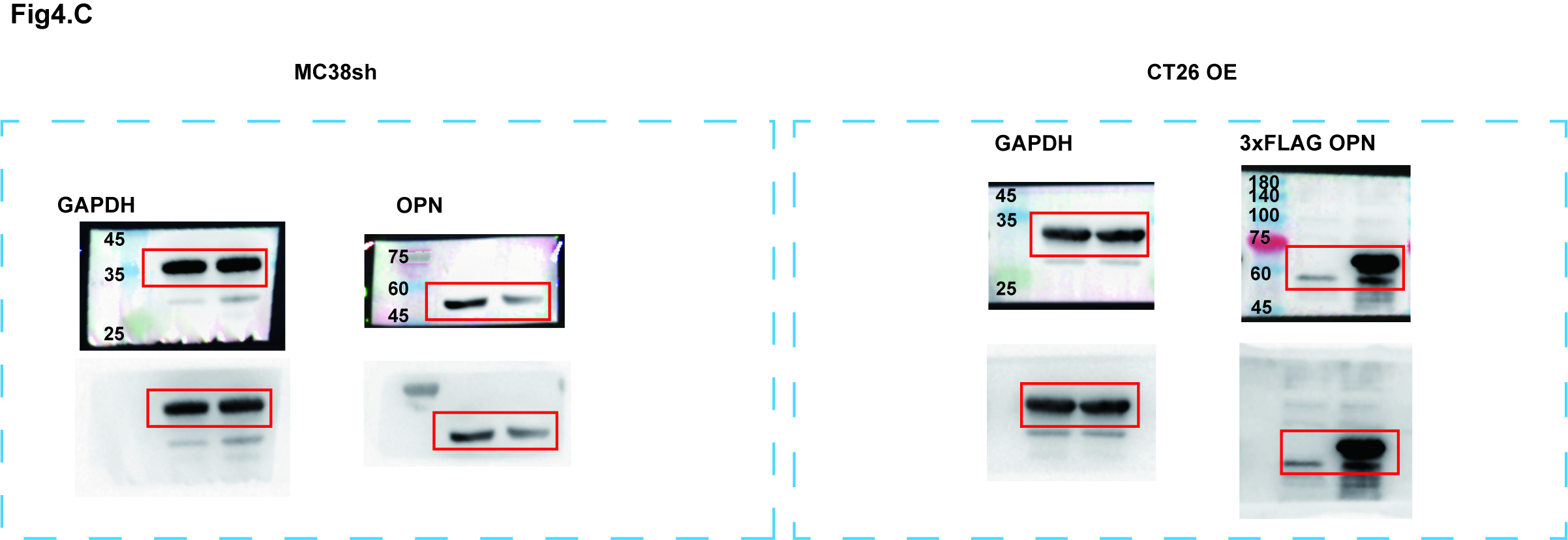

Supplement: Supplementary file 4 — Original WB Figure-4C [file 41420_2026_2945_MOESM4_ESM.tif]

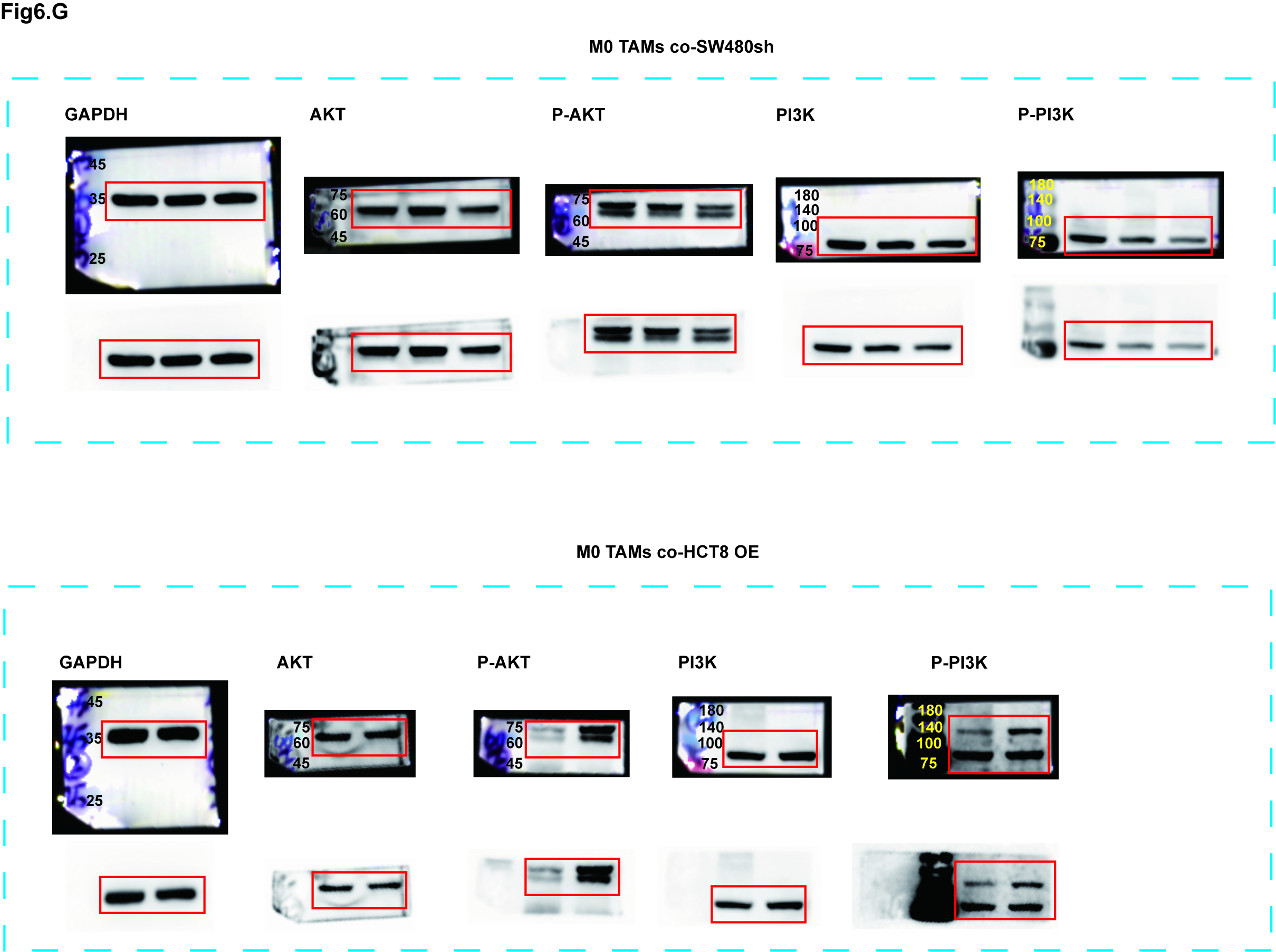

Supplement: Supplementary file 5 — Original WB Figure-6G [file 41420_2026_2945_MOESM5_ESM.tif]

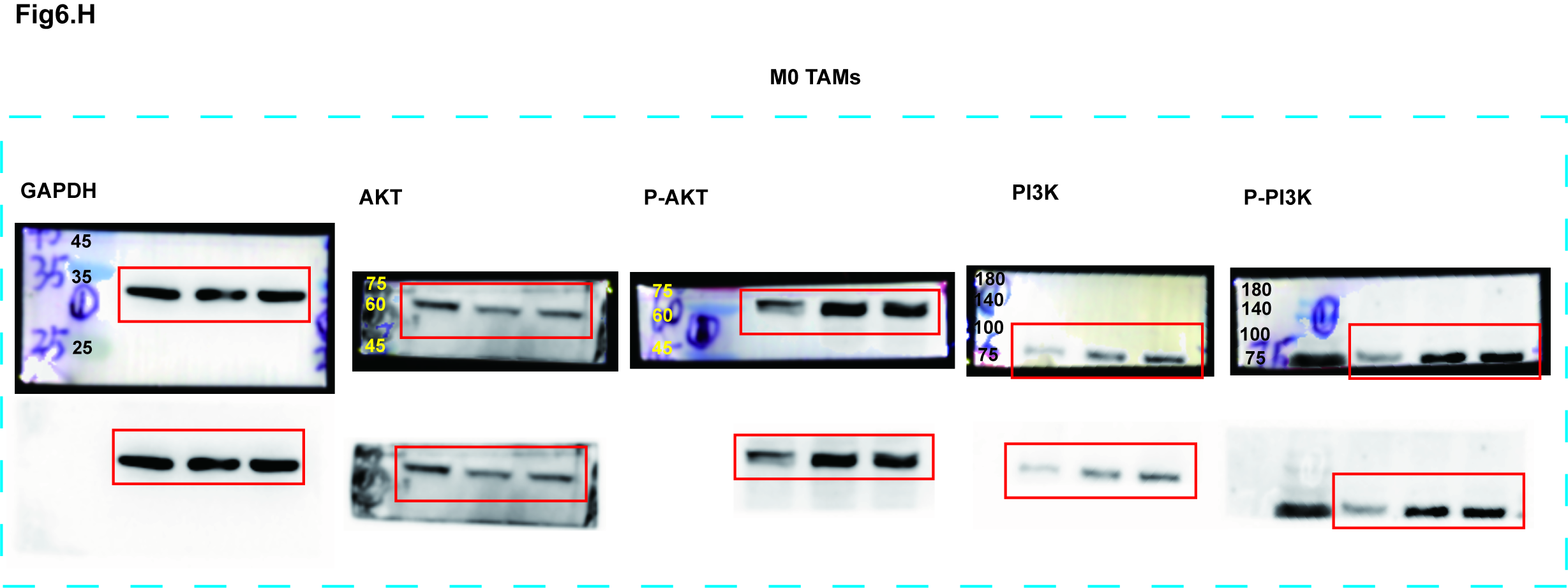

Supplement: Supplementary file 6 — Original WB Figure-6H [file 41420_2026_2945_MOESM6_ESM.tif]

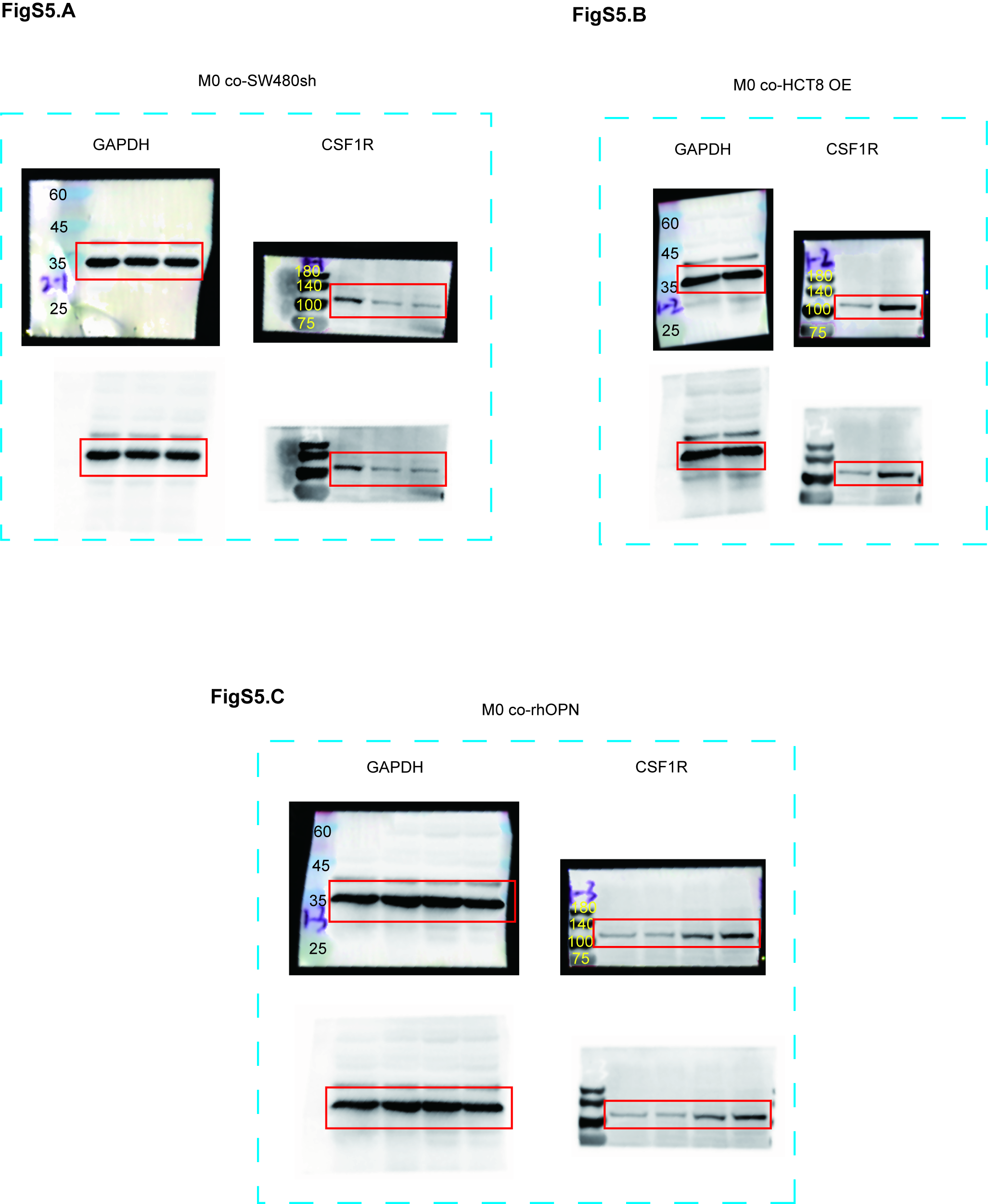

Supplement: Supplementary file 7 — Original WB Figure-S5A–C [file 41420_2026_2945_MOESM7_ESM.tif]

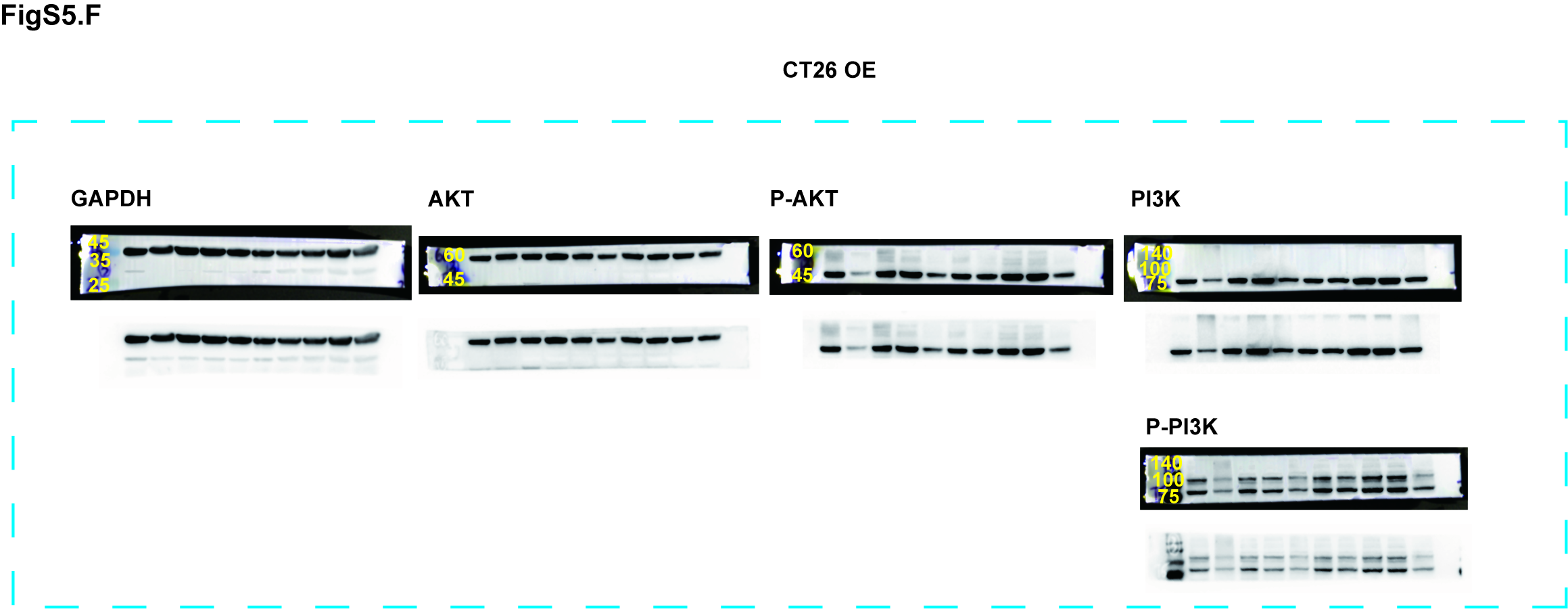

Supplement: Supplementary file 8 — Original WB Figure-S5F [file 41420_2026_2945_MOESM8_ESM.tif]
